# Assessment of water prophylaxis and biosafety in finfish aquaculture - current and emerging technologies

**DOI:** 10.3389/fmicb.2026.1841960

**Published:** 2026-08-04

**Authors:** Simona Bartkova, Francesco Valentino, Marcia Saraiva, Muhammed Duman, Anton Gerilovych, Vladimir Radosavljevic, Ario de Marco, Izzet Burcin Saticioglu, Hilal Ay

**Affiliations:** 1 Department of Chemistry and Biotechnology, Tallinn University of Technology, Tallinn, Estonia; 2 Department of Environmental Sciences, Informatics and Statistics, Ca’ Foscari University of Venice, Venice, Italy; 3 CIIMAR - Interdisciplinary Centre of Marine and Environmental Research, University of Porto, Matosinhos, Portugal; 4 Department of Aquatic Animal Diseases, Faculty of Veterinary Medicine, Bursa Uludag University, Bursa, Türkiye; 5 Institute for Problems of Cryobiology and Cryomedicine, National Academy of Sciences of Ukraine/One Health Scientific and Research Institute, PSI, Kharkiv, Ukraine; 6 Institute of Veterinary Medicine of Serbia, Belgrade, Serbia; 7 Laboratory of Environmental and Life Sciences, University of Nova Gorica, Nova Gorica, Slovenia; 8 Department of Molecular Biology and Genetics, Faculty of Arts and Science, Yildiz Technical University, Istanbul, Türkiye

**Keywords:** biosensors, finfish aquaculture, fish welfare, microfluidics, phage therapy, probiotics, water biosafety, water prophylaxis

## Abstract

Effective water quality management is a fundamental component of disease prevention, fish welfare, and sustainable production in finfish aquaculture. As aquaculture continues to expand to meet increasing global food demand, maintaining biosafe aquatic environments has become increasingly challenging due to production intensification, climate change, emerging pathogens, and the spread of antimicrobial resistance. While conventional approaches such as mechanical filtration, biofiltration, ultraviolet disinfection, ozonation, and water exchange remain central to water prophylaxis, increasing attention is being directed toward technologies that support earlier pathogen detection, improved risk assessment, and more targeted intervention strategies. This review examines water prophylaxis and biosafety through an integrated framework that links water quality, microbial ecology, pathogen surveillance, antimicrobial resistance, and preventive interventions. First, the biological and environmental mechanisms through which water quality influences disease susceptibility, microbiome stability, pathogen persistence, and antimicrobial resistance are evaluated. Conventional water-treatment technologies are then assessed alongside emerging approaches, including environmental DNA/environmental RNA monitoring, probiotics, phage therapy, biosensors, smart sensors, microfluidic platforms, and droplet-based molecular diagnostics. Emphasis is placed on their practical applicability, technological readiness, and suitability for different finfish production systems. The review highlights that effective biosafety management depends increasingly on integrating environmental monitoring with molecular diagnostics, risk assessment, and targeted interventions rather than relying solely on water-quality control or reactive disease treatment. Emerging technologies differ substantially in readiness, ranging from established and pilot-stage approaches to experimental technologies that require further validation before routine implementation. To support practical decision-making, the review synthesizes these technologies within an integrated water-prophylaxis and biosafety framework that links monitoring, diagnostics, risk assessment, intervention strategies, and reassessment pathways. Overall, sustainable finfish aquaculture will depend on combining conventional water-treatment infrastructure with advanced surveillance technologies and preventive biological interventions within coordinated biosafety programs. Such integration offers considerable potential to improve fish health, reduce environmental impacts, support responsible antimicrobial use, and enhance the long-term resilience of aquaculture production systems.

## 1 Introduction

**FIGURE 1:**
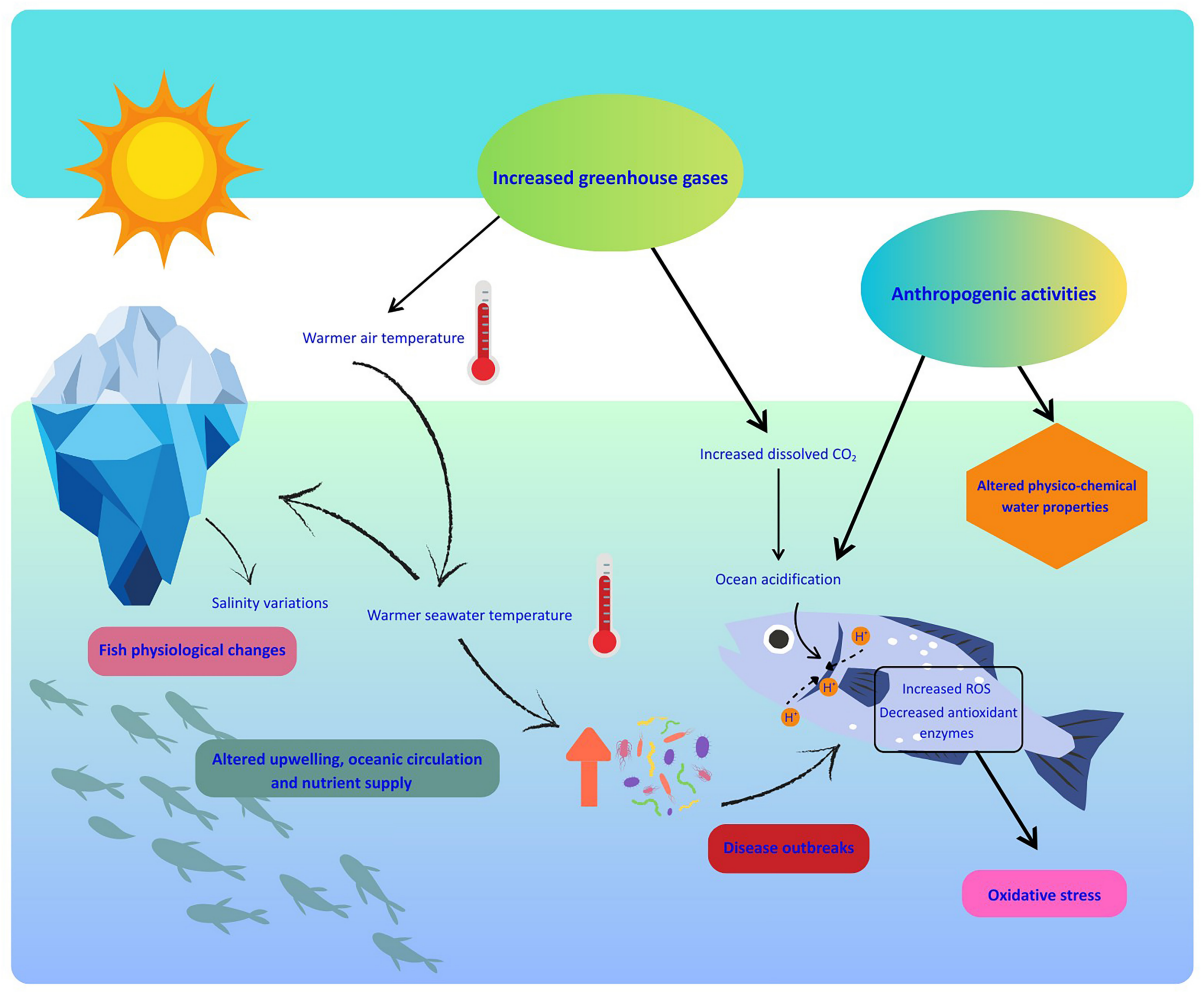
Schematic representation of key parameters influencing fish physiology and response to stressors. Temperature, salinity and pH shifts, caused by climate change and anthropogenic activities, can exacerbate disease outbreaks and physiological imbalances. Detailed information is provided in subsequent sections. Created in Canva. Saraiva, M. (2026).

**FIGURE 2:**
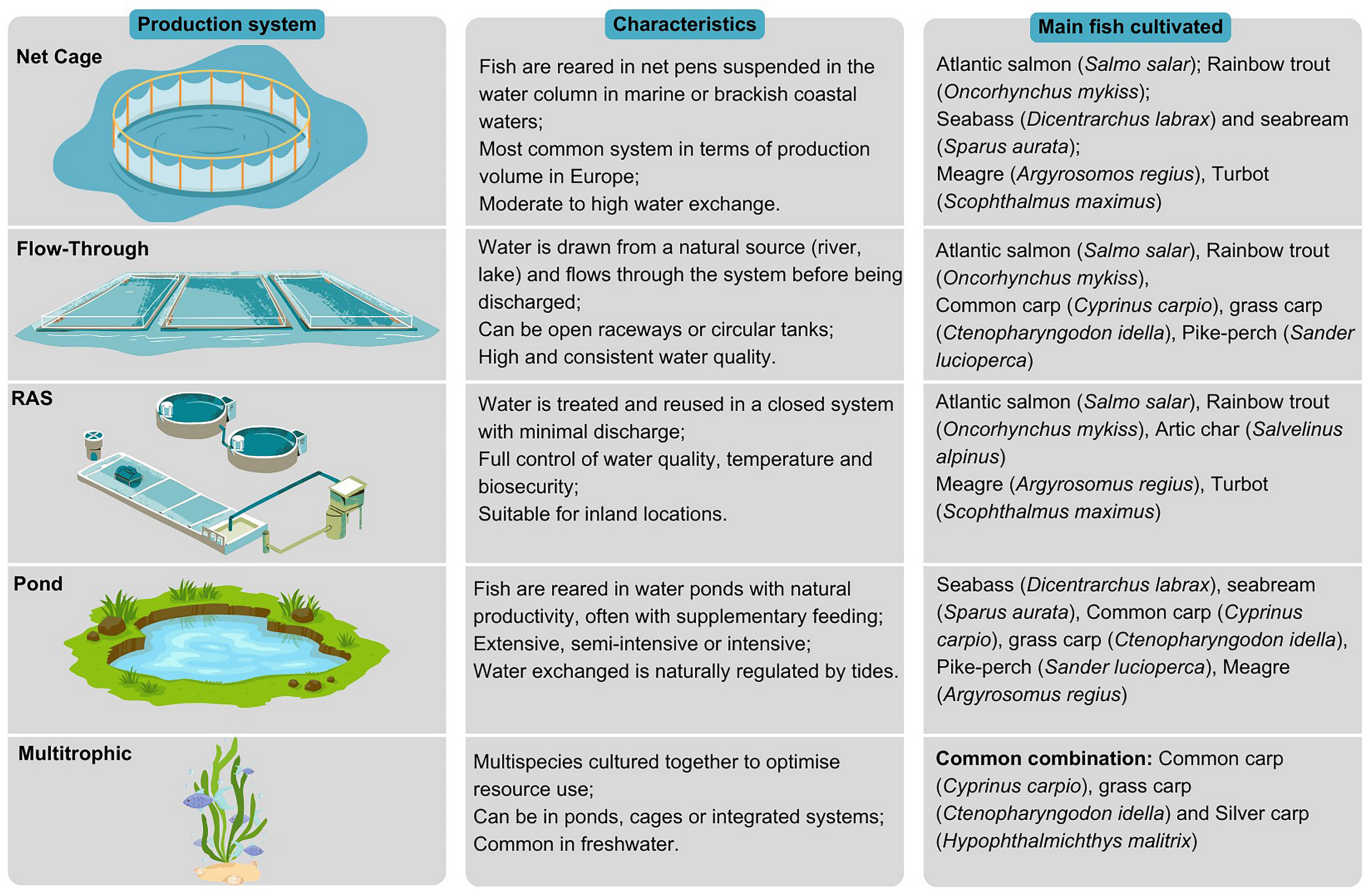
Overview of the principal finfish aquaculture systems practiced across Europe. The figure illustrates the main operational characteristics of each system and the fish species most commonly cultivated within them. It highlights the diversity of European aquaculture production methods, including marine cages, land-based flow-through systems, recirculating aquaculture systems (RAS), pond farming, and multitrophic systems, together with representative species such as Atlantic salmon, rainbow trout, European seabass, gilthead seabream, carp, and turbot. Created in Canva. Saraiva, M. (2026).

Water is the foundation of every aquaculture system, serving as the medium through which pathogens, microbial communities, and environmental stressors interact with cultured fish, making it a central determinant of health, productivity, and biosafety in aquaculture systems. While physicochemical parameters such as temperature, dissolved oxygen, pH, ammonia, salinity, and turbidity are traditionally used to assess water quality (You et al., 2021; Waller, 2024), their importance extends beyond fish physiology and production performance, governing feed efficiency, disease dynamics, and ultimately, the economic sustainability of production systems (Lindholm-Lehto, 2023; Tolentino et al., 2021). Fluctuations in these parameters can alter host immune function, influence microbial community structure, enhance pathogen persistence and virulence, and ultimately increase the risk of disease outbreaks. For example, low dissolved oxygen may exacerbate physiological stress and susceptibility to infection, while temperature changes can affect both pathogen replication and host defense mechanisms (Figure 1). Water quality management is therefore increasingly recognized not only as a production requirement but also as a fundamental component of disease prevention and biosafety in finfish aquaculture. Such interactions highlight why continuous monitoring and proactive management are more effective than reactive measures. However, the challenge of maintaining optimal water quality has intensified with the growth of intensive farming practices, climate change impacts, and the increasing emergence of aquatic pathogens, particularly in finfish aquaculture systems characterized by high stocking densities and strong physiological sensitivity to environmental change. Under these conditions, disease risk is shaped by a complex interaction between environmental conditions, microbial dynamics, and management practices. Although water quality is commonly assessed through physical, chemical, biological, and pathogen-related indicators (Su et al., 2020), their significance ultimately lies in their influence on microbial dynamics, pathogen persistence, and outbreak susceptibility. Because the environmental drivers of disease risk vary among production systems, the relative importance of water-quality parameters also differs across finfish farming systems (Figure 2). In pond aquaculture, commonly used in carp and northern pike farming in Central and Eastern Europe, as well as seabream and seabass farming in Portugal, factors such as pond age, fertilization, and stocking density strongly influence nutrient dynamics and algal blooms (Tziortzioti et al., 2019; Mramba and Kahindi, 2023). In recirculating aquaculture systems (RAS), widely applied to freshwater Atlantic salmon production, biofilter efficiency and water exchange rates are critical to controlling nitrogenous wastes (Boyd, 2017; Khanjani and Sharifinia, 2020). By contrast, cage culture in open water bodies, used for European seabass, Atlantic salmon, Gilthead seabream, Meager, Bluefin tuna, and Coho salmon farming, depends heavily on environmental conditions, which limit the farmer’s capacity to control water quality but increase the importance of site selection (Masser, 2012; Beveridge, 2004). Thus, although the general principles of water quality management are broadly applicable, their implementation and role in prevention and biosecurity require system-specific adaptations and integration with broader water prophylaxis and biosafety strategies. For example, inadequate water quality not only reduces productivity but also compromises immune responses, increasing vulnerability to bacterial and viral outbreaks in finfish populations (Shafi, 2025; Kumar et al., 2025; Combe et al., 2023). This highlights the importance of integrating water-quality management with broader surveillance and disease-prevention strategies.

**FIGURE 3:**
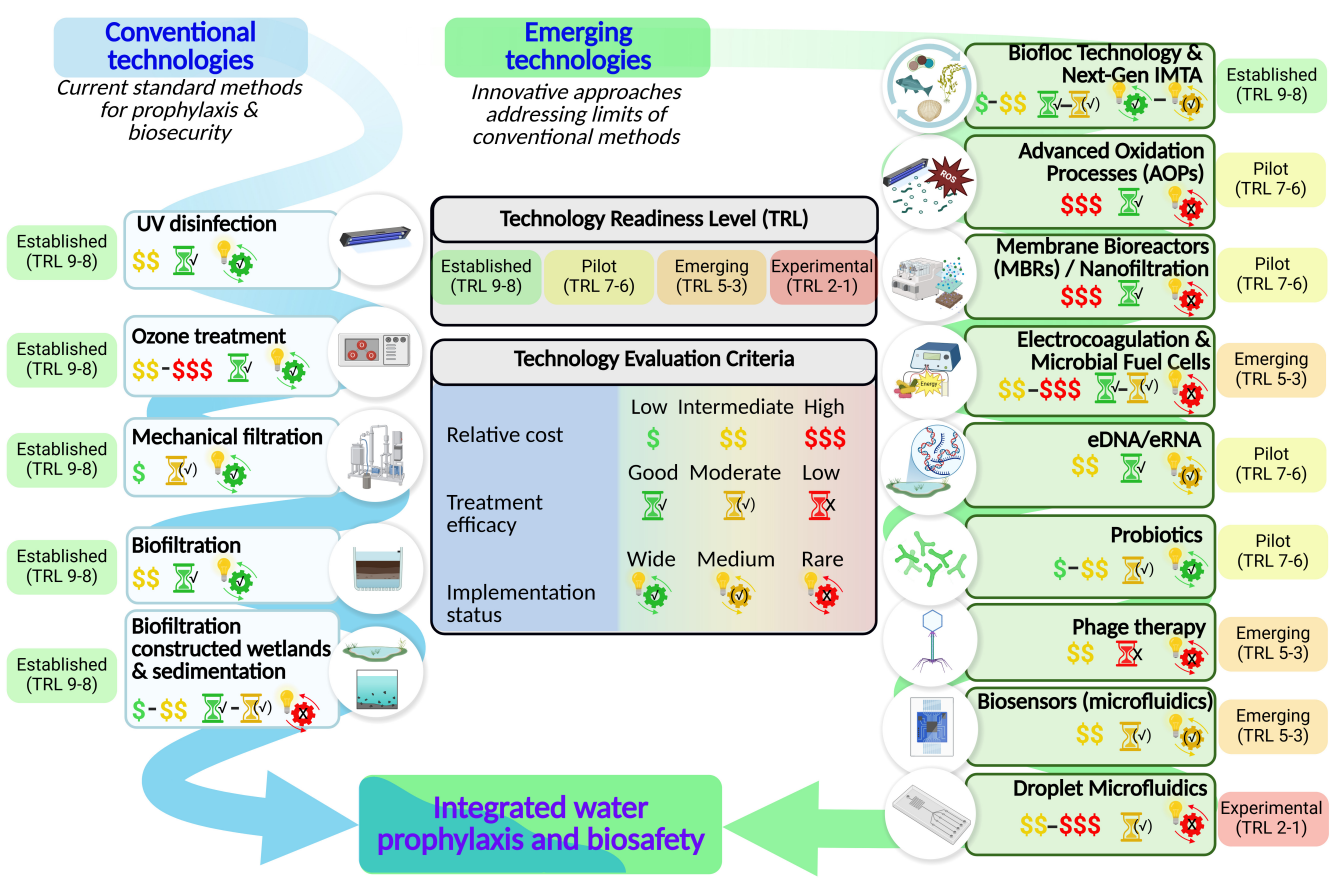
Conceptual decision-support pathway for water prophylaxis and biosafety management in finfish aquaculture. Monitoring signals related to water quality, microbial stability, pathogen abundance, and antimicrobial resistance are linked to diagnostic approaches, risk assessment, targeted interventions, and follow-up reassessment. The framework illustrates how conventional and emerging technologies discussed throughout this review (including eDNA/eRNA monitoring, ddPCR, microbial analyses, biosensors, microfluidics, probiotics, phage therapy, and water-treatment technologies), can be integrated into a coordinated biosafety management strategy across recirculating aquaculture systems (RAS), cage systems, and pond production systems. Created in BioRender. Bartkova, S. (2026) https://BioRender.com/fj95hef.

Recognizing water quality as a driver of pathogen emergence and transmission expands the role of water management beyond environmental control and into the broader domain of prophylaxis and biosafety. Water prophylaxis and biosafety encompass far more than the monitoring of physicochemical parameters. They include the management of microbial communities, identification of pathogen reservoirs, mitigation of antimicrobial resistance, implementation of preventive treatments, and deployment of technologies capable of detecting emerging threats before clinical disease occurs. Recent advances in molecular diagnostics, environmental DNA (eDNA) surveillance, biosensors, automated water-quality platforms, and data-driven surveillance systems have expanded the capacity for early pathogen detection and proactive intervention. These developments support a shift from reactive disease control toward preventive, risk-based management approaches that improve both system resilience and production sustainability. Accordingly, this review examines the interconnected roles of water quality, microbial ecology, pathogen surveillance, prophylactic interventions, and emerging monitoring technologies within a unified framework of water-based disease prevention in finfish aquaculture, with the aim of providing an integrated perspective on disease prevention and sustainable production. While the primary focus is on finfish systems, examples from other aquaculture sectors are included where they provide functional insights relevant to finfish aquaculture. Unlike previous reviews that have typically focused on individual topics such as water quality management, microbial ecology, probiotics, biosensors, or aquaculture biosafety, this review integrates these components within a unified water-prophylaxis and biosafety framework that links environmental monitoring, disease-risk assessment, preventive interventions, and emerging diagnostic technologies. The relationships among monitoring and surveillance, diagnostics, risk assessment, intervention strategies, and reassessment pathways discussed throughout this review are summarized in the integrated decision-support framework presented in Figure 3.

## 2 Importance of water quality parameters in aquaculture

### 2.1 Key water quality parameters

#### 2.1.1 Temperature

Temperature is one of the most influential environmental factors in aquaculture because it regulates metabolism, growth, reproduction, immune function, and pathogen development (Bowden et al., 2007; Whitney et al., 2016). Fish perform optimally within species-specific thermal ranges, whereas deviations from these optima can disrupt homeostasis, suppress immunity, alter microbiome composition, and increase susceptibility to infectious diseases (Le Morvan et al., 1998; Cascarano et al., 2021; Franke et al., 2024). In addition, temperature affects feeding behavior, aggression, and reproduction, while sudden fluctuations can trigger stress responses that reduce feed intake and growth performance (Cheng et al., 2009; Zhang et al., 2025). Early life stages are vulnerable because of their underdeveloped immune systems (Pepin, 1991; Zapata et al., 2006).

From a biosafety perspective, temperature influences both host susceptibility and pathogen virulence. Its effects are closely linked to other water quality parameters: elevated temperatures reduce dissolved oxygen availability while increasing metabolic oxygen demand, whereas lower temperatures may favor the persistence of certain parasitic infections. Consequently, temperature should be managed as part of an integrated water-quality and water-prophylaxis strategy rather than as an isolated parameter. Climate change is expected to increase thermal variability and associated disease risks, particularly for species cultured near their upper thermal limits (Agarwal et al., 2024). Adaptive strategies, including selective breeding, modified production systems, and predictive environmental management, are therefore becoming increasingly important for maintaining resilient finfish production (Ern et al., 2023; Jonsson, 2023).

#### 2.1.2 Dissolved oxygen

Dissolved oxygen (DO) is a critical determinant of fish welfare, metabolism, growth, and disease resistance (Farrell, 2009; Claireaux and Chabot, 2016). Because oxygen solubility decreases with increasing temperature and salinity, its availability is often a limiting factor in aquaculture systems (Timmons et al., 2018). Concentrations below 4 mg/L commonly impair growth, feed efficiency, and immune function, whereas severe hypoxia (<2 mg/L) can result in mass mortality events (Wu, 2002; Vaquer-Sunyer and Duarte, 2008; Ali and Mishra, 2022).

Dissolved oxygen is influenced by both environmental and management factors, including temperature, stocking density, feeding intensity, organic loading, and eutrophication (Díaz and Breitburg, 2009; Korpinen et al., 2015). Chronic oxygen depletion is especially important from a biosafety perspective because it compromises immune competence and increases susceptibility to opportunistic infections. Temperature and oxygen stress frequently act together, as elevated temperatures simultaneously increase oxygen demand while reducing oxygen availability.

The significance of DO varies among production systems. In ponds, daily fluctuations associated with photosynthesis and respiration can lead to nighttime hypoxia (Oberle et al., 2019). In cage culture, oxygen availability depends largely on natural water exchange and stratification, whereas RAS allow greater control through aeration and oxygenation technologies but require continuous monitoring to prevent system failures (Lindholm-Lehto, 2023). As climate-driven warming increases the frequency of hypoxic events (Zennaro et al., 2024), automated monitoring, predictive modeling, and emergency oxygenation systems are becoming increasingly important components of water prophylaxis and biosafety programs (Baena-Navarro et al., 2025; Yu M. et al., 2025).

#### 2.1.3 pH

Water pH is a key determinant of fish physiology, behavior, and survival, influencing acid-base balance, metabolic processes, and the toxicity of other waterborne compounds (Boyd and Tucker, 1998; Svobodová et al., 1993). Most finfish species tolerate pH values between 6.5 and 8.5, although species-specific sensitivities exist. Salmonids are generally more sensitive to elevated pH, whereas cyprinids such as carp and tench are vulnerable to extreme values, with pH levels above 10.8 or below 5.0 potentially causing mortality (Boyd, 2020; Svobodová et al., 1993). Even within nonlethal ranges, suboptimal pH can impair growth, reproduction, and immune function while increasing disease susceptibility (Boyd, 2020; Svobodová et al., 1993).

The biosafety importance of pH lies largely in its ability to modify the toxicity and bioavailability of compounds such as ammonia, hydrogen sulfide, cyanides, and heavy metals (Svobodová et al., 1993; Wang et al., 2019). In ponds, photosynthesis and respiration drive pronounced daily fluctuations, whereas in RAS, nitrification can reduce alkalinity and destabilize pH if buffering capacity is inadequate (Boyd, 2020; Ebeling et al., 2006; Malone and Pfeiffer, 2006). In cage systems, pH is influenced primarily by surrounding environmental conditions, including pollution and broader acidification processes (Doney et al., 2009; Holmer, 2010).

Maintaining stable pH conditions is therefore important not only for fish performance but also for preserving biofilter function and reducing pathogen-related risks. Climate change and increasing environmental acidification are expected to increase the need for real-time monitoring, predictive modeling, and adaptive buffering strategies in finfish aquaculture (Cabillon and Lazado, 2019; Tina et al., 2025; Cui et al., 2026).

#### 2.1.4 Ammonia and nitrite

Ammonia (NH_3_/NH_4_^+^) and nitrite (NO_2_^–^) are among the most important water quality indicators from a biosafety perspective because both directly impair fish health and increase disease susceptibility (Xu et al., 2021, 2022). These compounds originate primarily from fish excretion, protein catabolism, uneaten feed, and organic matter decomposition. Unionized ammonia (NH3) is particularly toxic, with toxicity increasing as temperature and pH rise (Kent, 2023). Even low concentrations can damage gill tissues, induce oxidative stress, suppress immune function, and increase vulnerability to infectious diseases (Wicks et al., 2002; Zhao et al., 2020). Sensitive species such as salmonids may be adversely affected at NH3 concentrations as low as 0.0125 mg/L (Timmons et al., 2018).

Nitrite, an intermediate product of nitrification, interferes with oxygen transport by converting hemoglobin to methemoglobin, causing the condition known as “brown blood disease” (Ajani et al., 2011). Concentrations above 0.5 mg/L are generally considered unsafe for many freshwater species, although tolerance varies among taxa and environmental conditions.

The accumulation of ammonia and nitrite is commonly associated with overfeeding, excessive stocking densities, inadequate water exchange, or failures in biological filtration. In ponds, nutrient accumulation and organic matter decomposition can lead to ammonia spikes during warm periods (Kumar et al., 2025). In RAS, biofilter dysfunction may result in rapid increases in ammonia and nitrite despite normally efficient nitrogen removal (Ruiz et al., 2020), whereas in cage culture, poor water circulation and localized waste accumulation may contribute to elevated nitrogenous waste levels (Gorlach-Lira et al., 2013). Because chronic exposure weakens immune responses and increases disease susceptibility, ammonia and nitrite should be regarded as priority indicators for routine biosafety monitoring. Effective control relies on optimized feeding, adequate water exchange, efficient biofiltration, and management of pH and temperature to minimize NH3 toxicity (Kumar et al., 2025). As climate change and eutrophication increase nitrogen-related stress, advanced monitoring and predictive management strategies will become increasingly important for maintaining fish health and system resilience (Ravikumar et al., 2024).

#### 2.1.5 Phosphorus

Unlike temperature, dissolved oxygen, pH, ammonia, and nitrite, phosphorus is not generally considered a direct biosafety parameter in finfish aquaculture. Instead, it functions primarily as an environmental loading indicator whose effects on fish health are mediated through ecosystem-level processes (Lall and Kaushik, 2021; Kibria et al., 2014).

Phosphorus is an essential nutrient required for growth, skeletal development, nucleic acid synthesis, energy metabolism, and feed efficiency. Deficiency can impair growth, feed utilization, and disease resistance, whereas adequate dietary supply supports normal physiological performance (Lall and Kaushik, 2021). However, excess dietary phosphorus is poorly retained and is largely excreted through feces, urine, and uneaten feed, contributing to phosphorus accumulation in aquaculture effluents (Kibria et al., 2014).

The biosafety relevance of phosphorus lies primarily in its contribution to eutrophication. Elevated phosphorus concentrations promote algal blooms, increase organic loading, and contribute to oxygen depletion, creating environmental conditions that can compromise fish welfare and increase disease risk (Varol and Balcı, 2020; Mavraganis et al., 2021; Brenckman et al., 2025). Together with nitrogen compounds, phosphorus is a major driver of eutrophication and ecosystem instability in both freshwater and coastal systems (Wurtsbaugh et al., 2019).

Therefore, phosphorus should be viewed primarily as an environmental pressure factor rather than a direct physiological stressor. Effective management focuses on reducing nutrient discharge, improving feed efficiency, and limiting downstream ecological impacts that may indirectly affect fish health and biosafety (Hejazy et al., 2023).

#### 2.1.6 Turbidity

Turbidity refers to water cloudiness caused by suspended particles such as silt, clay, organic matter, plankton, and uneaten feed residues, and it is an important indicator of water quality in aquaculture systems (Davies-Colley and Smith, 2001; Tziortzioti et al., 2019). High turbidity can reduce feeding efficiency by limiting visual prey detection, impair gill function through irritation or clogging, and reduce light penetration, affecting primary productivity and ecosystem stability (Zhang et al., 2025). From a biosafety perspective, turbidity also matters because suspended organic matter can support microbial growth and provide shelter for pathogens and parasites, increasing infection pressure (Pulkkinen et al., 2010). In addition, it can reduce the effectiveness of UV treatment and chemical oxidants by shielding microorganisms from exposure (Hijnen et al., 2006).

The factors contributing to turbidity, and their management, vary among aquaculture systems. In ponds, it often results from sediment resuspension, phytoplankton blooms, and high stocking densities (Boyd, 2020). In cage aquaculture, river inflows, currents, and seasonal runoff can increase suspended solids beyond farm control (Holmer, 2010). In RAS, turbidity is usually controlled through mechanical and biological filtration, but system failures or inadequate solids removal can rapidly degrade water quality (Schumann and Brinker, 2020). Effective turbidity management therefore supports both production efficiency and biosafety by reducing pathogen persistence and improving water treatment performance. Control strategies include optimized feeding, improved solids removal, enhanced filtration in RAS, and careful management of external water inputs in open systems.

#### 2.1.7 Salinity

Salinity, defined as the concentration of dissolved salts in water, is a key regulator of osmotic balance and physiological performance in aquatic organisms (Boeuf and Payan, 2001; Varsamos et al., 2005). Fish and invertebrates are adapted to specific salinity ranges, and deviations from these conditions can induce osmotic stress, impair growth and immune function, and, in extreme cases, cause mortality. From a biosafety perspective, salinity influences both host physiology and pathogen dynamics. It can modify the toxicity of other water quality parameters; for example, ammonia toxicity generally decreases with increasing salinity, whereas the bioavailability and toxicity of certain metals and pollutants may increase under specific conditions (Hall et al., 1998). In addition, many aquatic pathogens exhibit salinity-dependent survival, growth, and infectivity, linking salinity fluctuations to disease occurrence and transmission dynamics (Pulkkinen et al., 2010).

The importance of salinity varies among aquaculture systems. Freshwater species cultured in ponds and RAS are sensitive to salinity changes caused by evaporation, water source variability, or salt intrusion (Wedemeyer, 1996). In marine and brackish-water systems, fluctuations driven by rainfall, tides, and freshwater inflows may stress cultured species, especially during early developmental stages (Boeuf and Payan, 2001). Cage systems are fully exposed to ambient salinity variability, whereas RAS provide greater opportunities for salinity control through water mixing and supplementation (Badiola et al., 2012). Climate change is expected to increase salinity instability through altered freshwater availability, increased evaporation, and saltwater intrusion into coastal and estuarine environments (Zennaro et al., 2024; IPCC, 2019). Consequently, continuous monitoring and system-specific salinity management remain important components of water prophylaxis and biosafety programs.

#### 2.1.8 Comparative significance of water quality parameters for biosafety

Together, water quality parameters influence fish health and ecosystem sustainability, although their effects on disease susceptibility and biosafety differ. Temperature and dissolved oxygen have broad effects because they directly regulate metabolism, immune function, pathogen virulence, and environmental carrying capacity (Farrell, 2009; Claireaux and Chabot, 2016; Le Morvan et al., 1998; Cascarano et al., 2021; Wu, 2002). Ammonia and nitrite serve as key indicators of system performance, reflecting the effectiveness of feeding practices, waste management, water exchange, and biofiltration, while also directly affecting fish health and disease susceptibility (Xu et al., 2021, 2022; Zhao et al., 2020). pH further shapes biological responses by modifying the toxicity of ammonia and other dissolved compounds, thereby influencing the impact of multiple environmental stressors (Svobodová et al., 1993; Wang et al., 2019). Turbidity and salinity affect pathogen persistence, treatment efficacy, physiological resilience, and host-environment interactions (Pulkkinen et al., 2010; Hijnen et al., 2006; Boeuf and Payan, 2001), whereas phosphorus contributes more indirectly through eutrophication, harmful algal blooms, and ecosystem destabilization (Varol and Balcı, 2020; Wurtsbaugh et al., 2019). Therefore, effective water prophylaxis requires not only monitoring individual parameters but also considering their interactions and relative significance within different aquaculture systems. A summary of these parameters, their biological effects, system-specific considerations, and biosafety implications is provided in Table 1. Together, these factors form the environmental foundation upon which disease risk, surveillance priorities, and intervention strategies are subsequently assessed.

**TABLE 1:** Water quality thresholds, biological effects, disease risks, and management considerations in finfish aquaculture systems [adapted from Boyd (2020), Timmons et al. (2018), Xiao et al. (2019)].

Parameter	Optimal range (general finfish)	Sublethal stress threshold	Critical/lethal threshold	Key biological effects	System-specific risks (Pond/Cage/RAS)	Biosafety implications	Relationship to disease susceptibility	Monitoring and management considerations
Temperature (°C)	Species-specific (e.g., carp: 20 °C–28 °C; salmonids: 10 °C–16 °C)	±3 °C–5 °C deviation from optimum; rapid shifts stressful	Beyond species tolerance limits → mortality	Regulates metabolism, immune function, pathogen virulence, behavior, and microbiome composition	Pond: Seasonal and diurnal fluctuation Cage: Limited control and climate variability RAS: High control but vulnerable to system or power failure	Thermal stress suppresses immunity and promotes pathogen proliferation	Influences immunity, microbiome stability, pathogen virulence, and dissolved oxygen availability	Continuous monitoring is widely available; management depends strongly on system type
Dissolved oxygen (DO) (mg/L)	>5–6 mg/L	3–5 mg/L	<2 mg/L → hypoxia; <1 mg/L → lethal	Supports respiration, feeding, metabolism, and immune function	Pond: Nocturnal hypoxia Cage: Stratification and low flow RAS: Aeration or system failure	Low DO impairs metabolism and immunity, increasing infection risk	Low oxygen levels increase stress and susceptibility to bacterial and parasitic infections	Early warning parameter; requires reliable aeration and backup
pH	6.5–8.5	5.5–6.5 or 8.5–9.5	<5.0 or >10.5	Affects ion balance, toxicity, reproduction, and physiological stability	Pond: Diel fluctuation; Cage: Environmental exposure RAS: Acidification from nitrification	pH shifts increase toxicity and alter pathogen virulence, elevating stress and susceptibility	Affects immune function, pathogen dynamics, and the toxicity of other compounds	Easily monitored; management relies on buffering capacity and water chemistry control
Unionized ammonia (NH_3_)	<0.02 mg/L	0.02–0.05 mg/L	>0.05–0.2 mg/L	Causes gill damage, oxidative stress, and immunosuppression	Pond: Feeding and organic load spikes Cage: Poor flushing RAS: Biofilter failure	Highly toxic at low levels	Damages gills and suppresses immunity, facilitating infection	Requires strict biofiltration and feeding control; routine monitoring is well established
Nitrite (NO_2_^–^)	<0.1 mg/L	0.1–0.5 mg/L	>0.5–1 mg/L	Causes methemoglobinemia and reduced oxygen transport	Pond: System imbalance Cage: Accumulation under low exchange RAS: Nitrification instability	Reduces oxygen transport and increases physiological stress, raising disease risk	Impairs oxygen transport and increases physiological stress, thereby increasing susceptibility to opportunistic infections	Requires monitoring and mitigation (e.g., chloride)
Nitrate (NO_3_^–^)	<50 mg/L	50–100 mg/L	>100–300 mg/L	Associated with reduced growth and reproductive performance	RAS: Accumulation Pond/Cage: Dilution via exchange	Indicates chronic water quality deterioration	Chronic exposure may impair physiological performance and increase disease susceptibility through prolonged stress	Requires dilution or denitrification; easily monitored and managed through solids removal and filtration
Phosphorus (mg/L)	<0.05 mg/L	0.05–0.1 mg/L	>0.1 mg/L	Promotes algal blooms and oxygen depletion	Pond: Eutrophication; Cage: Nutrient loading RAS: Effluent accumulation	Drives eutrophication and bloom formation, leading to hypoxia and disease risk	Contributes indirectly to disease risk through eutrophication, harmful algal blooms, and oxygen depletion	Easily monitored, although management options vary among production systems
Turbidity (NTU)	<25	25–80	>100	Impairs feeding, irritates gills, and provides pathogen refuge	Pond: Sediment resuspension Cage: External inflow RAS: Filtration failure	Enhances pathogen persistence and reduces disinfection efficiency	Elevated turbidity may increase pathogen persistence and infection pressure by reducing water quality and treatment efficacy	Requires filtration and input management
Salinity (ppt)	Species-specific (FW < 0.5; marine 30–35)	Rapid fluctuation (>2–5 ppt)	Outside tolerance → mortality	Regulates osmoregulation, metabolism, and pathogen dynamics	Cage: Environmental variability RAS: Controlled conditions Pond: Evaporation effects	Fluctuations disrupt osmotic balance and influence pathogen survival	Stable conditions reduce stress and disease risk	Continuous monitoring is widely available; management depends strongly on system type

### 2.2 Fish microbiome and water-host interactions

Over the past decade, the study of the fish microbiome has evolved from a largely descriptive field into a rapidly expanding area of integrative biology, with increasing emphasis on the functional understanding of host-microbe interactions. This shift has been driven by advances in sequencing technologies and the development of experimental model systems. Fish, as aquatic vertebrates, harbor complex and dynamic microbial communities across multiple body sites, most notably the gut, skin, and gills. Unlike terrestrial animals, their microbiomes are in constant interaction with the surrounding water, making them sensitive to environmental conditions and microbial exchange (Bisai et al., 2026; Chen et al., 2022). Among these microbial habitats, the gut microbiome is the most extensively studied due to its central role in host physiology, including digestion (Jiao et al., 2023), immunity (El-Son et al., 2025), and development (Yukgehnaish et al., 2020).

It is now well established that fish gut communities are dominated by bacteria, especially members of the Proteobacteria and, to a lesser extent, Firmicutes. This composition contrasts from that of mammals, where Firmicutes and Bacteroidetes typically prevail (Kim et al., 2021). To date, the gut microbiota has been evaluated in a few model fish species, such as zebrafish (Roeselers et al., 2011), guppy (Sullam et al., 2012), and rainbow trout (Navarrete et al., 2010), and in economically valuable aquatic animals, such as carp (Ye et al., 2013), Atlantic salmon (Hovda et al., 2007), sturgeon (Geraylou et al., 2012), and Atlantic cod (Wilson et al., 2008). However, only a small fraction of the more than 30,000 known fish species has been studied, highlighting a considerable gap in taxonomic and ecological coverage (Soh et al., 2024).

Beyond bacteria, fish microbiomes also include archaea, fungi, protists, and viruses, although these components remain comparatively understudied (Singh et al., 2025; Guirado-Flores et al., 2025). Growing evidence suggests that these non-bacterial members may contribute considerably to host functions, including digestion and immune modulation; however, their precise roles are still being elucidated (Llewellyn et al., 2014; Raggi et al., 2020).

The microbiome begins to establish early in life, with colonization occurring soon after hatching through exposure to the egg surface, water, and first feeding, and continues to develop dynamically throughout the fish life cycle (Uniacke-Lowe et al., 2024; Uddin et al., 2026). Functionally, the fish microbiome is now recognized as an integral component of host biology. It contributes to digestion, nutrient assimilation, and energy metabolism, while also playing a critical role in immune regulation and disease resistance. Recent evidence shows that microbiota influences mucosal system development, epithelial growth, and overall physiological performance (Ma et al., 2025; Wu Z. et al., 2025). From a biosafety perspective, the microbiome represents both a defensive barrier against pathogen invasion and a sensitive indicator of environmental disturbance. Resident microbial communities contribute to colonization resistance through competition for nutrients and attachment sites, production of antimicrobial compounds, and modulation of host immune responses (Llewellyn et al., 2014; Kelly and Salinas, 2017; Butt and Volkoff, 2019). Conversely, disruption of these communities can facilitate pathogen establishment and increase disease susceptibility in both wild and farmed fish (Ihuţ et al., 2026). As a result, maintaining microbiome stability is increasingly regarded as a complementary component of water prophylaxis and disease prevention strategies in aquaculture systems, reinforcing their importance in host health.

A central insight from recent research is that fish microbiome composition is shaped by a complex interaction among environmental conditions, host characteristics, and management practices. Among these factors, water quality and surrounding microbial communities exert strong influences because fish continuously exchange microorganisms with their aquatic environment (Sullam et al., 2012; Tarnecki et al., 2017; Bisai et al., 2026). Water characteristics, including salinity, temperature, water chemistry, and microbial composition, strongly shape gut microbial communities. Comparative studies between marine and freshwater fish consistently show distinct microbiome structures, emphasizing the important role of environmental context (Singh et al., 2025). However, host genotype, developmental stage, stocking density, and husbandry practices also contribute substantially to microbiome assembly and function, often interacting with water chemistry and microbial exposure (Tarnecki et al., 2017; Butt and Volkoff, 2019; Uddin et al., 2026). Diet is another major determinant, with feeding strategies influencing microbial diversity and metabolic potential. Experimental studies, such as those on tilapia, demonstrate that natural environments with more diverse diets support higher microbial diversity compared to controlled aquaculture systems (Ghazali et al., 2025). Moreover, environmental stressors, including pollution, temperature shifts, and salinity changes, can significantly alter microbiome composition and function (Yoon et al., 2026; Zhou et al., 2025).

These insights have important implications for aquaculture, where the fish microbiome is increasingly viewed not only as a determinant for improving productivity and sustainability but also as a target for disease prevention and biosafety management. Manipulation of microbial communities through probiotics, prebiotics, and dietary strategies has shown promise in enhancing growth, feed efficiency, and disease resistance (Diwan et al., 2024). Simultaneously, the microbiome is being explored as a source of novel antimicrobial compounds, such as bacteriocins, with potential applications in both aquaculture and human health (Uniacke-Lowe et al., 2024). Despite these advances, a key challenge remains the lack of a clear definition of what constitutes a “healthy” microbiome, limiting the ability to standardize interventions across species and production systems (Tay et al., 2025). Microbiome “health” has been described using multiple criteria, including taxonomic diversity, temporal stability, resilience to environmental disturbances, functional redundancy, pathogen exclusion capacity, and positive effects on host growth and immune performance (Llewellyn et al., 2014; Butt and Volkoff, 2019; Banerjee and Ray, 2017). However, these dimensions are not always equivalent, as taxonomically distinct communities may fulfill similar ecological functions. This complicates the identification of reliable microbiome-based indicators of health, posing a major challenge for the design and evaluation of prophylactic strategies in aquaculture. Increasing evidence now supports viewing a “healthy” microbiome as a dynamic functional continuum, shaped less by fixed taxonomic composition and more by its microbial diversity, functional resilience, and preservation of key ecological roles. Functions such as nutrient metabolism, immune modulation, and defense against pathogen colonization are emerging as more robust and informative indicators of health across species than the mere presence or absence of specific microbial taxa (Ihuţ et al., 2026; Lorgen-Ritchie et al., 2023).

Methodologically, the field has benefited from rapid technological progress. High-throughput sequencing, metagenomics, and other omics approaches have enabled more comprehensive characterization of microbial communities, including previously unculturable taxa. These tools are now facilitating a shift toward functional microbiomics, allowing researchers to investigate not only which microorganisms are present but also their metabolic roles and interactions within the host (Kanika et al., 2025). In aquaculture, metagenomics, transcriptomics, metabolomics, and other integrated omics approaches are increasingly being explored for early-warning surveillance, biomarker discovery, pathogen-risk assessment, and the design of targeted microbiome-based interventions (Tarnecki et al., 2017; Minich et al., 2020). Such approaches have considerable potential to support proactive biosafety management by detecting microbial shifts associated with environmental stress, dysbiosis, or emerging disease threats before clinical signs become evident.

Despite this progress, major knowledge gaps persist. The ecological roles of non-bacterial microbiota remain poorly characterized, and distinguishing stable resident microbes from transient environmental taxa continues to be challenging. Furthermore, many studies remain correlational, with limited experimental work to establish causal relationships between microbiome composition and host traits. Addressing these limitations will be essential for advancing the field toward predictive and mechanistic understanding.

In summary, the fish microbiome is a highly dynamic, environmentally driven system that is closely linked to host nutrition, immunity, and development. An important mechanism linking water quality to disease susceptibility, and therefore to water prophylaxis and biosafety in finfish aquaculture, is microbiome dysbiosis, defined as a disruption of host-associated microbial communities. Environmental stressors such as ammonia, hypoxia, and temperature fluctuations can alter both water and mucosal microbiota (gut, gill, and skin), reducing diversity and destabilizing host–microbe interactions, which may increase susceptibility to opportunistic pathogens (Llewellyn et al., 2014; Sylvain et al., 2016). These processes are shaped by continuous microbial exchange between water and host surfaces, linking system-level environmental conditions to host-associated microbial structure and function (Dehler et al., 2017). Consequently, monitoring microbiome dynamics may provide an additional layer of bio-surveillance, enabling earlier detection of deteriorating environmental conditions and elevated disease risk than conventional physicochemical indicators alone. While significant progress has been made, further work is required to develop a more predictive and mechanistic understanding of these processes in aquaculture systems.

### 2.3 Relationship between water quality and disease dynamics

Water quality is a major determinant of disease susceptibility in aquaculture systems. Suboptimal conditions act as chronic stressors that weaken immune defenses and increase vulnerability to infectious agents, including viruses, bacteria, fungi/oomycetes, and parasites (Ciji and Akhtar, 2021). At the same time, poor water quality can promote pathogen persistence, virulence, and transmission, creating conditions that favor disease outbreaks (Liu et al., 2025). These effects arise from both environmental factors, such as temperature fluctuations, eutrophication, and organic matter accumulation, and management-related factors, including overfeeding, inadequate waste removal, and insufficient water treatment. For example, biofilms developing in tanks, pipelines, and water supply systems can serve as reservoirs for pathogenic *Aeromonas*, *Flavobacterium*, and *Vibrio* species, facilitating recurrent infection cycles (King et al., 2004; Cai et al., 2013). Similarly, suspended organic matter from uneaten feed, feces, and decomposing material may protect viral particles from inactivation and prolong their persistence in the aquatic environment (Wu H. et al., 2025).

The relationship between water quality and disease dynamics is highly system specific. In ponds, stagnant areas and sediment accumulation favor pathogen persistence (Boyd, 1995). In RAS, inadequate sanitation can allow biofilms to develop within biofilters and distribution networks (Al-Harbi et al., 2025). Cage systems are influenced by ambient environmental conditions, including plankton blooms and temperature fluctuations, which may trigger stress-related disease outbreaks (Holmer, 2010). Consequently, water quality management should be regarded not only as a production requirement but also as a core component of aquaculture biosafety. Key water quality parameters and their influence on disease dynamics across different production systems are summarized in Table 1. These interactions form the basis of risk-based monitoring and intervention strategies, whereby changes in water quality, microbial stability, pathogen abundance, or antimicrobial resistance indicators can trigger targeted management responses, as summarized in the decision-support framework presented in Figure 3.

### 2.4 Aquaculture water as a reservoir of pathogens

Aquatic environments serve as both reservoirs and transmission routes for a wide range of pathogens, including bacteria, viruses, fungi/oomycetes, protozoa, and helminths (Amarasiri et al., 2021). Water also supports disease vectors and intermediate hosts such as mollusks, copepods, and aquatic insects, which can facilitate pathogen survival and dissemination (Hung and Remais, 2008; Fornillos et al., 2019). As a result, aquaculture water represents a critical interface linking animal health, environmental health, and biosafety.

Recent studies have shown that aquaculture operations can contribute to pathogen dissemination beyond farm boundaries. eDNA analyses have detected pathogen spillover from net-pen salmon farms to surrounding wild populations (Bass et al., 2024), while modeling studies demonstrate that waterborne pathogens may spread between farms depending on hydrological conditions and farm location (Oidtmann et al., 2014; Roh and Kannimuthu, 2023). Viral pathogens are of especially concerning, with novel aquatic RNA viruses and circoviruses continuing to emerge in aquaculture systems (Ahmadivand et al., 2025; Wang et al., 2025).

Open and semi-open production systems are especially vulnerable to contamination from upstream sources, including agricultural runoff, untreated effluents, biotechnological waste, and wild or farmed animal carriers. In these systems, water functions both as a reservoir and a conduit for pathogen transmission, enabling the spread of infectious agents within and between farms. Shared rivers, lakes, and coastal waters therefore represent significant biosecurity risks, as pathogens from multiple sources can accumulate and persist (Bouwmeester et al., 2021). Protozoan parasites are also increasingly detected in aquatic environments, including waters associated with aquaculture production and shellfish intended for human consumption, highlighting the potential for zoonotic transmission (Duan et al., 2025; Suarez et al., 2025).

Several factors increase infection pressure, including high stocking densities, limited water exchange, inadequate filtration, and the accumulation of organic matter. Effective mitigation therefore requires a combination of water treatment, controlled water intake, routine pathogen surveillance, and system-specific biosecurity measures (Shafi, 2025; El-Hack et al., 2022). These approaches reduce pathogen loads, limit opportunities for inter-farm transmission, and improve resilience to disease outbreaks (Liu et al., 2025). Biosecurity measures applied in different finfish aquaculture systems, together with their applications and limitations, are summarized in Table 2. Their integration within broader biosafety decision-making frameworks is illustrated in Figure 3.

**TABLE 2:** Water-mediated pathogen dynamics in finfish aquaculture systems, representative pathogens, transmission pathways, and biosecurity implications [adapted from Subasinghe et al. (2023), Shafi (2025), El-Hack et al. (2022)].

Aquaculture system	Pathogen presence (representative pathogens)	Transmission pathways	System-specific risks	Biosecurity implications and mitigation strategies
Open pond systems	Bacteria: *Aeromonas hydrophila*, *Aeromonas salmonicida*, *Flavobacterium columnare* Viruses: CyHV-3, CEV Fungi/Oomycetes: *Saprolegnia* spp. Protozoa: *Ichthyophthirius multifiliis* Helminths: *Digenean trematodes*	Direct exposure to surface waters, runoff, wild fish, birds, and invertebrate vectors	High exposure to diverse environmental pathogens and sediment reservoirs	Continuous water quality monitoring; pathogen surveillance; bioexclusion of wild fauna; controlled water exchange; sediment management
Semi-open systems (raceways, flow-through)	Bacteria: *Yersinia ruckeri*, *Aeromonas* spp., *Flavobacterium psychrophilum* Viruses: IHNV, VHSV, IPNV Protozoa: *Ichthyobodo necator*, *Trichodina* spp.	Contaminated influent water; effluent backflow; upstream farm connectivity	Medium-high risk of pathogen introduction from upstream and recirculation	Multi-barrier water treatment (filtration, UV/ozonation); surveillance programs; intake risk assessment; effluent control
Recirculating aquaculture systems (RAS)	Bacteria: *Aeromonas* spp., *Pseudomonas* spp., *Mycobacterium* spp. Biofilm-associated: *Flavobacterium* spp. Other: Opportunistic microbial complexes	Endogenous amplification via biofilms; organic loading System failures	Internal pathogen build-up due to biofilms and recirculation issues	Biofilter integrity; biofilm control; disinfection; microbial surveillance; strict hygiene
Cage culture (lakes, rivers, marine/coastal)	Bacteria: *Aeromonas salmonicida*, *Vibrio anguillarum* Viruses: ISAV, VHSV, IHNV Parasites: *Gyrodactylus salaris*, *Ichthyophthirius multifiliis* Other: Plankton-associated opportunists	Continuous exposure to ambient water, hydrodynamics, plankton blooms, and wildlife	High environmental exposure and dispersal risks	Site selection; stocking density control; health surveillance; ecosystem monitoring
Shared water bodies/watershed-connected systems	Multi-host pathogens: *Aeromonas* spp., *Vibrio* spp., CyHV-3, VHSV, CEV Other: Wildlife reservoirs and persistent agents	Hydrological connectivity; wildlife spread; farm-to-farm transmission	Regional-scale pathogen persistence and spread	Coordinated governance and buffer zones; synchronized disease control and watershed surveillance
Live fish transport systems	Bacteria: *Aeromonas salmonicida*, *Yersinia ruckeri* Viruses: IHNV, VHSV, CyHV-3 Parasites: *Ichthyophthirius multifiliis*, *Gyrodactylus* spp.	Movement of infected or subclinical fish; stress-induced shedding	High risk of disease introduction between locations	Quarantine, health certification; transport disinfection; stress reduction; traceability

### 2.5 Aquaculture water as reservoir of antimicrobial resistance genes

Aquatic environments associated with finfish aquaculture act as important reservoirs and transmission interfaces for antimicrobial resistance (AMR), linking animal, environmental, and human health within a One Health framework. Intensive production systems, high stocking densities, and the historical use of antimicrobials create conditions that favor the selection, persistence, and dissemination of antimicrobial-resistant bacteria (ARB) and antimicrobial resistance genes (ARGs) (Preena et al., 2020; Milijasevic et al., 2024; Deng et al., 2025). Within aquaculture systems, ARGs represent an additional biosafety concern because they may contribute to the persistence and dissemination of resistant microorganisms. These dynamics may also have implications for human health through environmental dissemination pathways and the potential transfer of resistant bacteria via aquaculture effluents and products.

Antimicrobial resistance genes in aquaculture systems are frequently associated with mobile genetic elements, including plasmids, transposons, and integrons, which facilitate horizontal gene transfer (HGT) across taxonomically diverse microbial communities. Especially class 1 integrons, widely recognized as key drivers of multidrug resistance, are commonly detected in aquaculture sediments, biofilms, and water columns, often carrying gene cassettes conferring resistance to sulfonamides, tetracyclines, and β-lactams (Fouz et al., 2020; Goh et al., 2024). Plasmid-mediated resistance, including transferable genes encoding extended-spectrum β-lactamases (ESBLs) and quinolone resistance, further enhances the dissemination potential of AMR within aquaculture-associated microbiomes. These mobile elements are not confined to pathogenic bacteria but are widely distributed among environmental and commensal taxa, creating a persistent reservoir of resistance determinants that can be mobilized under selective pressure. The co-occurrence of heavy metals and biocides in aquaculture environments may further promote co-selection of resistance traits, amplifying the persistence of ARGs even in the absence of direct antibiotic exposure (Barcan et al., 2025).

The detection of ARGs does not necessarily indicate phenotypic resistance, nor does it directly imply transfer to clinically relevant pathogens. These represent distinct aspects of AMR risk. ARG abundance reflects the environmental resistance reservoir, phenotypic resistance reflects the expression of resistance traits within microbial communities, whereas transfer risk depends on the presence of mobile genetic elements and opportunities for horizontal gene transfer. Among the commonly monitored indicators of ARG contamination in aquaculture water are class 1 integrons and sul1, together with resistance determinants such as tetA and qnr, which are frequently detected in aquaculture environments and associated with mobile resistance elements (Fouz et al., 2020; Goh et al., 2024).

The prevalence and distribution of ARGs vary significantly across aquaculture system types. Open and semi-open systems (e.g., ponds and cage cultures) are vulnerable to external inputs of resistance determinants from agricultural runoff, wastewater discharge, and wildlife vectors. In such systems, ARG abundance in water and sediment can reach levels comparable to those observed in impacted natural ecosystems, with reported concentrations of *sul1*, *tetA*, and *qnr* genes frequently exceeding 10^4^–10^6^ gene copies per mL or gram in heavily impacted environments (Fouz et al., 2020; Deng et al., 2025). In contrast, RAS present a different risk profile: although external inputs are reduced, internal recirculation promotes the accumulation and persistence of ARGs within biofilters, tank biofilms, and sludge. Studies have shown that biofilter-associated microbial communities can act as hotspots for ARG enrichment, with significantly higher ARG diversity and abundance compared to incoming water (Goh et al., 2024). These system-specific differences highlight the need for tailored AMR risk assessment and management strategies.

Within these systems, biofilms represent an important microenvironment influencing AMR dynamics. Biofilms formed on tank surfaces, pipelines, and biofilter media facilitate horizontal gene transfer through high cell density, close physical proximity, and the presence of extracellular polymeric substances. These conditions enhance the efficiency of conjugation, transformation, and transduction processes. Experimental and environmental studies suggest that HGT rates in biofilms can be substantially higher (up to 10–1000×) than in planktonic phases, although estimates vary among microbial communities, environmental conditions, and experimental systems (Roh and Kannimuthu, 2023; Al-Harbi et al., 2025). In finfish aquaculture systems, biofilms are ubiquitous and difficult to eliminate, especially in RAS and hatchery environments. Their persistence enables long-term retention of ARGs and resistant bacteria, even in the absence of ongoing antimicrobial use, thereby contributing to the resilience of AMR within aquaculture ecosystems.

Addressing AMR is therefore an important component of water prophylaxis and biosafety. Probiotics, prebiotics, and synbiotics can reduce pathogen load and limit ecological niches available to resistant bacteria, thereby indirectly lowering selection pressure for ARGs. However, their application must be carefully evaluated to avoid introducing strains carrying transferable resistance genes. The presence of ARGs within a probiotic strain does not necessarily preclude its use; however, transferable resistance determinants and mobile genetic elements represent biosafety concerns. Consequently, probiotic candidates should undergo strain-level characterization, including screening for transferable resistance determinants and mobile genetic elements, before application in aquaculture systems (Hoseinifar et al., 2018). Phage therapy represents a promising “green” alternative to antibiotics by enabling highly specific targeting of major finfish bacterial pathogens, including *Aeromonas* spp. (e.g., *A. salmonicida* and *A. hydrophila*), *Vibrio* spp. (e.g., *V. anguillarum* and *V. alginolyticus*), and biofilm-associated opportunists such as *Flavobacterium* spp., which are recurrent causes of disease outbreaks in intensive aquaculture (Żaczek et al., 2020).

At the system level, management strategies such as reducing organic load, optimizing water exchange, and controlling biofilm formation are critical for limiting ARG persistence and horizontal gene transfer. Advanced treatment technologies, including UV irradiation, ozonation, and membrane filtration, may further reduce ARG abundance, although their effectiveness depends on factors such as gene form (intracellular versus extracellular DNA) and environmental conditions. The main sources, transmission pathways, system-specific risks, and potential mitigation strategies for ARGs in aquaculture water are summarized in Table 3. Within integrated biosafety frameworks, ARG monitoring and risk assessment provide an important basis for selecting appropriate mitigation and intervention strategies (Figure 3).

**TABLE 3:** Antimicrobial resistance genes (ARGs) in aquaculture water, including antimicrobial resistance (AMR) sources, transmission pathways, system-specific risks, and biosecurity implications and mitigation strategies [adapted from Fouz et al. (2020), Goh et al. (2024), Deng et al. (2025)].

AMR source	Transmission pathways	Aquaculture system-specific risks	Biosecurity implications and mitigation strategies
Hospital and municipal wastewater	Discharge introduces resistant bacteria and genes into aquatic environments	Open/semi-open ponds: Direct contamination from upstream water	Water intake control; routine surveillance; effluent treatment
Agricultural runoff	Antibiotic residues and resistant bacteria carried into ponds or cages	Ponds/cages near farms: Nutrient- and antibiotic-laden inflows	Buffer zones; runoff management; periodic water quality testing
Aquaculture effluents	Residual antibiotics select for resistance within microbial communities	Ponds/cages: Sediment and water column RAS: Biofilter accumulation	Optimize feeding; reduce antibiotic use; regular cleaning and biofilter maintenance
Wildlife and human carriers	Fish, birds, and other wild and domestic animals introduce resistant microbes	Open ponds/cages: Exposed to wildlife RAS: Less affected but internal contamination possible	Physical barriers; quarantine; hygiene measures; wildlife deterrents
Internal aquaculture microbiota	Horizontal gene transfer between microbial communities	RAS and tanks: Biofilm-associated AMR propagation	Biofilm control; disinfection; microbial community management; ARG surveillance
Long-distance fish transport	Transmission of resistant bacteria with livestock	All systems: Receiving transported fish	Quarantine; health certification; transport hygiene; pathogen surveillance

### 2.6 Regulatory and biosecurity considerations

Water quality is increasingly recognized as a central component of aquaculture biosecurity programs. Regulatory frameworks at both national and international levels emphasize water management as a means of preventing disease outbreaks, ensuring animal welfare, and maintaining environmental sustainability (Subasinghe et al., 2023). Consequently, water quality management is no longer viewed solely as a production requirement but also as a key element of risk-based disease prevention, water prophylaxis, and biosafety planning.

Effective biosecurity programs must address the entire water cycle within aquaculture systems. This includes securing water sources free of pathogens, pollutants, and ARGs; applying appropriate physical, chemical, and biological treatment processes such as filtration, UV irradiation, ozonation, and biofiltration; maintaining water quality during recirculation and reuse; and managing effluents to minimize nutrient loading and pathogen release into surrounding environments. Together, these measures reduce infection pressure within production systems while limiting pathogen transmission and ARG dissemination to natural water bodies (Chu et al., 2025; World Health Organization [WHO], 2025).

The relative importance of individual measures varies among production systems. Open and semi-open systems are vulnerable to pathogen introduction through incoming water, environmental runoff, and wildlife vectors, whereas RAS place greater emphasis on water treatment, biofilter performance, sanitation, and continuous monitoring. As aquaculture intensification increases, integrating water quality management with broader biosecurity planning becomes increasingly important for maintaining both animal health and environmental protection.

Sustainable aquaculture therefore depends on regulatory compliance combined with proactive biosecurity practices, including routine monitoring, risk assessment, contingency planning, and system-specific management strategies (Milijasevic et al., 2024; Subasinghe et al., 2023). Regulatory frameworks, key biosecurity measures, and system-specific considerations for aquaculture water management are summarized in Table 4 and their integration within a broader biosafety programs is illustrated in Figure 3.

**TABLE 4:** Interrelationships among aquaculture water-management regulatory frameworks, key requirements, biosecurity measures, and system-specific considerations [adapted from Subasinghe et al. (2023), Milijasevic et al. (2024), Chu et al. (2025)].

Regulatory frameworks	Key requirements	Biosecurity measures	System-specific considerations
Water sourcing	Ensure pathogen-free, uncontaminated water (national/international standards)	Source testing; surveillance for pathogens and ARGs	Ponds: Upstream contamination risk RAS: Controlled intake Cages: Ambient water monitoring
Water treatment	Physical, chemical, and biological treatments are recommended	Filtration; UV; ozonation; biofiltration; chlorination (where allowed)	RAS: Biofilter efficiency Ponds/cages: Pre-treatment of inflow water
Recirculation and reuse	Minimize pathogen proliferation while reusing water	Regular monitoring; disinfection; sediment removal; biofilter maintenance	RAS: Continuous quality control Ponds: Partial reuse Cages: Limited control
Effluent and discharge management	Uphold environmental protection standards; control nutrient and pathogen load	Settling ponds; filtration; controlled release; monitoring nutrient levels	Ponds/cages: Direct discharge risk RAS: Treated effluent reuse or controlled discharge
Monitoring and contingency	Routine water-quality monitoring and pathogen surveillance; risk assessment	Regular sampling; early warning systems; emergency response plans	Applies across all systems; higher frequency in intensive and shared water bodies
Integrated biosecurity planning	Include water management as a core component	SOPs for water handling, hygiene, quarantine, and disease-outbreak response	Tailored to system type, stocking density, and environmental exposure

## 3 Conventional technologies for water quality management

### 3.1 Principles and system-specific considerations

Conventional water treatment technologies remain the foundation of water prophylaxis and biosafety in finfish aquaculture. However, these technologies should not be viewed as interchangeable solutions but rather as complementary components of a system-specific treatment train. Their effectiveness depends on production system design, organic and particulate loading, turbidity, hydraulic retention time, operational capacity, and biosafety objectives. Consequently, technology selection should be guided not only by treatment performance but also by implementation feasibility, operational complexity, and system-specific risks.

The continued expansion and intensification of aquaculture have increased the need for effective water treatment strategies capable of maintaining animal health while reducing environmental impacts (Food and Agriculture Organization of the United Nations [FAO], 2022; Cojocaru et al., 2022; Bjørndal et al., 2024). In intensive finfish systems, particularly land-based and RAS, the accumulation of organic matter, nitrogenous wastes, residual chemicals, and microbial contaminants can compromise both production efficiency and biosafety (Hamdhani et al., 2020; González-Gaya et al., 2022). Effective treatment therefore serves a dual purpose: maintaining water quality within production systems and reducing the release of pollutants and pathogens into surrounding environments (Zhang et al., 2006).

The technologies most applied in finfish aquaculture include mechanical filtration, biofiltration, UV disinfection, ozonation, and, where appropriate, nature-based polishing systems such as constructed wetlands and sedimentation units (Tom et al., 2021). Although these approaches are generally considered established technologies, their suitability depends on production system characteristics and treatment objectives. Mechanical filtration and biofiltration are important in intensive finfish operations with high organic and nitrogenous loading, whereas constructed wetlands are more commonly used for effluent polishing and nutrient reduction (Zhou et al., 2014; Tom et al., 2021).

While the primary focus of this review is finfish aquaculture, differences among aquaculture sectors should be recognized. High-load treatment approaches such as biofiltration, biofloc systems, and membrane bioreactors are most relevant to intensive finfish production, whereas shellfish systems often rely more heavily on natural filtration processes and generally generate lower particulate and nutrient loads (Zimmermann et al., 2023). Similarly, seaweed cultivation functions largely as a nutrient extraction system rather than a major source of water pollution, shifting management priorities from wastewater treatment toward biosafety and environmental monitoring (Campbell et al., 2019). These differences highlight the importance of tailoring water treatment strategies to specific production systems rather than applying a single approach across all aquaculture sectors.

**FIGURE 4:**
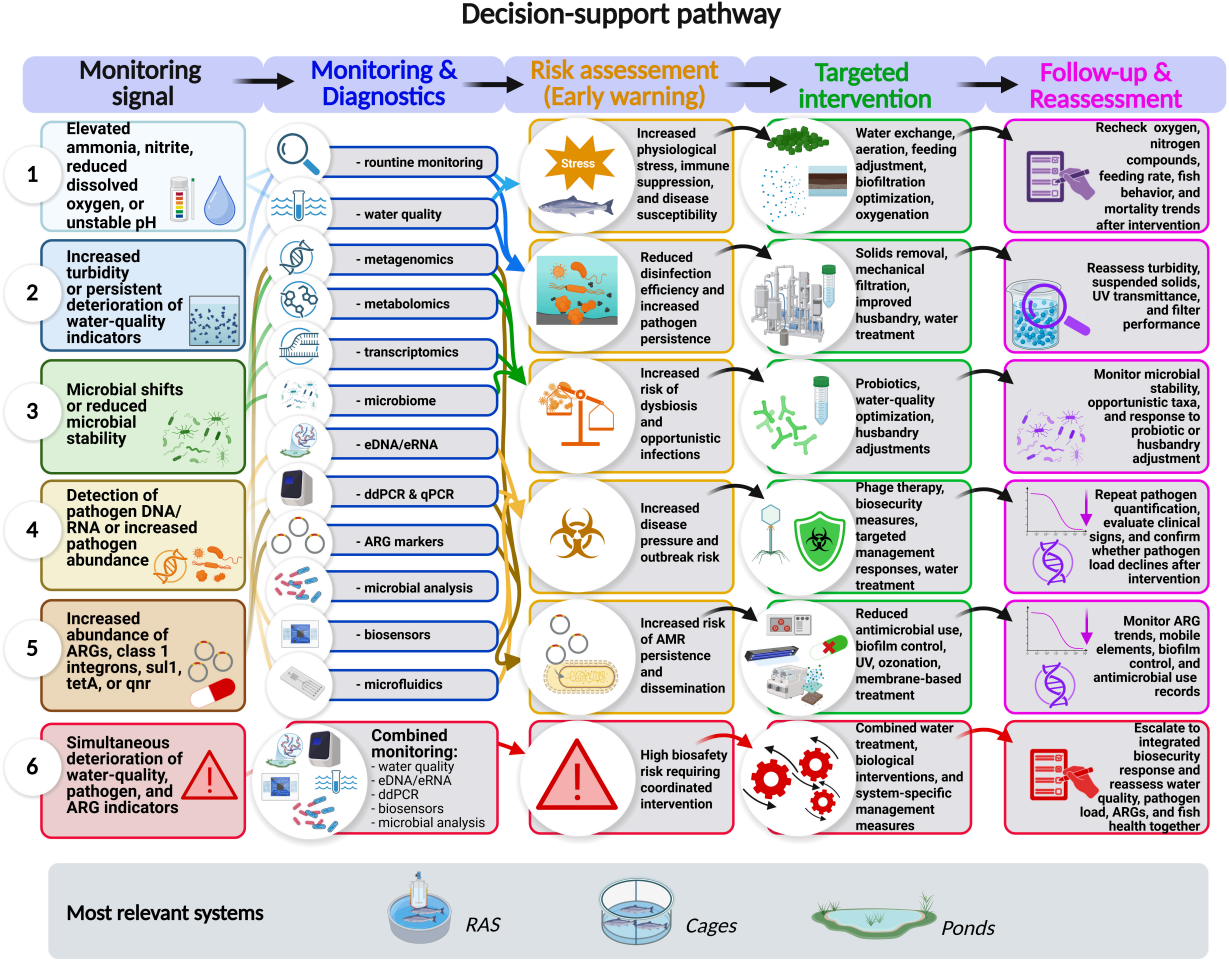
Comparative evaluation of conventional and emerging technologies for water prophylaxis and biosafety in finfish aquaculture. Conventional technologies represent established treatment approaches widely implemented in finfish production systems, particularly in land-based and recirculating aquaculture systems (RAS), including mechanical filtration, biofiltration, UV disinfection, ozone treatment, and, where applicable, constructed wetlands and sedimentation. These technologies form the foundation of water treatment strategies by supporting solids removal, nitrogen transformation, and microbial control. Emerging technologies, including advanced oxidation processes (AOPs), membrane bioreactors (MBRs), electrocoagulation, microbial fuel cells (MFCs), biofloc technology, next-generation integrated multi-trophic aquaculture (IMTA), phage therapy, probiotics, biosensors, microfluidics, and environmental DNA/RNA (eDNA/eRNA) monitoring, aim to address limitations of conventional systems by enhancing contaminant removal, improving resource utilization, and supporting precision monitoring and disease prevention. Technologies are classified according to technology readiness level (TRL), ranging from established (TRL 9-8) to pilot (TRL 7-6), emerging (TRL 5-3), and experimental (TRL 2-1) approaches. Technologies are further evaluated according to relative cost, treatment efficacy, and implementation status. Icons indicate relative cost ($–$$$), treatment efficacy (high, moderate, or low), and implementation status (widely implemented, system-dependent, or emerging/experimental). Classifications are qualitative and may vary according to production system, operational scale, regulatory framework, and environmental conditions. Together, conventional and emerging technologies contribute to integrated water prophylaxis and biosafety strategies in finfish aquaculture. Created with BioRender. Bartkova, S. (2026) https://BioRender.com/a5xpyo0.

The relative position of conventional technologies within the broader water-prophylaxis and biosafety landscape, together with selected emerging approaches, is summarized in Figure 4. Because conventional and emerging technologies differ substantially in cost, treatment efficacy, and implementation requirements, Figure 4 provides a comparative overview of conventional and emerging technologies used for water prophylaxis and biosafety in finfish aquaculture. Technologies are categorized according to technology readiness level (TRL) and qualitatively compared based on relative cost, treatment efficacy, and implementation status.

No single technology is sufficient to ensure water quality and biosafety under all production conditions. Instead, effective water management relies on integrating solids removal, biological treatment, and disinfection processes within a system-specific treatment train. The optimal configuration depends on production intensity, water source, pathogen risk, and operational constraints.

The following sections evaluate conventional technologies not only by their treatment mechanisms but also by their strengths, limitations, likely failure modes, and suitability for different finfish production systems (Xiao et al., 2019). While these technologies remain essential components of water prophylaxis and biosafety, emerging approaches increasingly complement them through enhanced monitoring, surveillance, diagnostics, and preventive interventions.

### 3.2 UV disinfection

Ultraviolet (UV) disinfection is one of the most widely implemented water treatment technologies in aquaculture and is classified as an established technology with high implementation readiness (TRL 9-8; Figure 4). UV-C irradiation (200–280 nm) provides chemical-free microbial control by inactivating microorganisms through DNA damage that prevents replication (Adeniyi and Jimoh, 2024). This approach is important for reducing waterborne microbial loads, including contaminants such as *Escherichia coli* and *Pseudomonas aeruginosa*, which may compromise aquatic animal health and pose downstream public health concerns (Mena and Gerba, 2009). Recent developments suggest that UV performance can be enhanced through coupling with TiO_2_-based photocatalysis, which generates reactive oxygen species (ROS) that broaden antimicrobial activity (Ahmad et al., 2022; Lang et al., 2022).

In finfish RAS, UV treatment is typically applied as a downstream or side-stream barrier to reduce microbial loads in recirculating water without introducing chemical residues (Xiao et al., 2019). Reported reductions in culturable bacterial counts can be substantial, and emerging configurations such as mercury-free UV-LED systems combined with peroxymonosulfate aim to improve treatment efficiency while reducing energy consumption (Tom et al., 2021). However, UV efficacy is strongly influenced by water UV transmittance and turbidity, making effective upstream solids removal essential in intensive finfish systems. UV may also be less effective against certain resistant pathogens, including *Cryptosporidium parvum* oocysts and *Legionella pneumophila*, supporting the use of integrated treatment approaches in high-risk systems (Craik et al., 2001).

Beyond pathogen control, UV treatment aligns with sustainable aquaculture objectives by avoiding chemical accumulation and reducing selection pressure associated with antimicrobial use (Huyben et al., 2018; Lang et al., 2022; Collivignarelli et al., 2021; Zhu et al., 2025). UV-based processes may also contribute to the removal of contaminants of emerging concern, including pharmaceuticals and hormones (Wijekoon et al., 2013). Overall, UV is most effective as a polishing and disinfection step in hatcheries and RAS where turbidity and suspended solids are already controlled. Its principal advantages are residue-free microbial reduction and high implementation readiness, whereas limitations include reduced performance under high turbidity, lamp fouling, and insufficient exposure time. Consequently, UV should be integrated with effective solids removal and routine monitoring of UV transmittance rather than used as a stand-alone biosafety barrier.

### 3.3 Ozone treatment

Ozone treatment is another established water treatment technology (TRL 9-8; Figure 4) widely used to improve water quality in intensive finfish production systems and RAS (Masjidin et al., 2024). As a strong oxidizing agent, ozone rapidly transforms a broad range of pollutants, including dissolved organic carbon, nitrite, pharmaceuticals, and microbial contaminants, while improving water clarity, odor, and coloration (Davidson et al., 2011; Powell and Scolding, 2018). These properties make ozonation valuable in systems characterized by high organic and nutrient loading (Aich et al., 2020).

In finfish RAS, ozonation has been associated with improved operational performance through reductions in fine particulates and dissolved organic matter, thereby enhancing subsequent treatment processes such as biofiltration (Aich et al., 2020; Guan et al., 2014). Ozone can also degrade residual pharmaceutical compounds, including hormones such as 17β-estradiol and antibiotics that may persist in aquaculture effluents (Schroeder et al., 2011; Pumkaew et al., 2021). Together with UV treatment, ozonation contributes to reducing waterborne loads of opportunistic bacterial pathogens frequently associated with disease outbreaks, including *Aeromonas* spp., *Vibrio* spp., *Lactococcus* spp., *Yersinia ruckeri*, *Tenacibaculum* spp., *Photobacterium* spp., and *Flavobacterium* spp.

The main strengths of ozone treatment are its broad-spectrum oxidation capacity, improvement of water clarity, and support of downstream disinfection and biofiltration processes. However, residual ozone and oxidation by-products may adversely affect fish and beneficial biofilter communities if treatment is not carefully controlled. For this reason, ozonation requires monitoring of oxidation-reduction potential, contact time, off-gassing, and species-specific tolerance. When appropriately managed, ozone remains a valuable component of integrated water treatment trains designed to improve biosafety, water reuse, and environmental sustainability (Gerrity and Snyder, 2011).

### 3.4 Biofiltration

Biofiltration remains a cornerstone technology for maintaining water quality in finfish RAS and is considered an established treatment approach with high implementation readiness (TRL 9-8; Figure 4; Godzieba et al., 2025; Abdelfatah et al., 2022). Conventional biofilters convert toxic ammonia excreted by cultured fish into nitrate through sequential nitrification, thereby reducing one of the primary water-quality risks in intensive production systems. However, nitrate accumulation, acidification, and competition for dissolved oxygen between nitrifying microorganisms and cultured fish require careful management (Navada et al., 2020; Ramli et al., 2020; Gao et al., 2020; Ebeling et al., 2006).

Recent developments have focused on improving biofilter stability and enabling more complete nitrogen removal through optimized media selection and staged nitrification-denitrification designs (Yang et al., 2001). Media such as plastic Pall Rings™, crushed coke, and zeolite can enhance biofilm development and ammonia removal, while integrated aerobic-anoxic systems improve nitrogen transformation and reduce nitrate accumulation (Mnyoro et al., 2022; Cahill et al., 2010; Tao and Chang, 2025; Trivedi, 2009; Carboni et al., 2022). Functional specialization within biofilms is reflected by the dominance of *Nitrosomonas* and *Nitrospira* during nitrification and *Thiobacillus*-associated taxa during denitrification, supporting stable nitrogen cycling and improved pH regulation (Fei et al., 2020).

From a biosafety perspective, biofilters should be regarded as living treatment systems rather than passive infrastructure. Their performance depends on biofilm maturity, oxygen availability, alkalinity, temperature, pH, and hydraulic loading. Sudden changes in stocking density, feeding intensity, disinfectant exposure, or antibiotic use may destabilize microbial communities and lead to ammonia or nitrite accumulation. Maintaining biofilter stability is therefore critical not only for water quality management but also for water prophylaxis and disease prevention in intensive finfish production systems.

### 3.5 Mechanical filtration

Mechanical filtration is the first control point in most water treatment trains and remains one of the most widely implemented technologies in finfish aquaculture (TRL 9-8; Figure 4; Tawfik et al., 2023; Roy et al., 2025; Xiao et al., 2019). By removing suspended solids and particulate organic matter derived from uneaten feed, feces, and biofloc, mechanical filtration reduces oxygen demand and improves the performance of downstream treatment processes, including biofiltration, UV disinfection, and ozonation (Schumann and Brinker, 2020; Lyu et al., 2024).

A wide range of technologies are available, including swirl separators, hydrocyclones, microscreen drum filters, sand filters, parabolic screen filters (PSFs), foam fractionators, and protein skimmers (Zezulka et al., 2024; Rushton et al., 2008). Selection depends on particle size distribution, operational requirements, and system design. Coarse solids are commonly removed using swirl separators and hydrocyclones (Chenglin et al., 2015), whereas drum filters and sand filters are widely applied for finer particulate removal (Davidson et al., 2013; Yu et al., 2014). PSFs provide passive coarse filtration (Xiao et al., 2019; Buffle et al., 2019), while foam fractionators and protein skimmers are mostly useful in marine finfish systems for removing fine particulates and dissolved organic compounds while improving gas exchange (Sochacki et al., 2025; Gregersen et al., 2021; Waller, 2024; Kovács et al., 2023; Buckley et al., 2022; Rahman et al., 2012).

No single mechanical approach is sufficient across all particulate fractions. Modern finfish RAS therefore increasingly rely on integrated filtration systems that combine multiple technologies to maximize solids removal while balancing efficiency, energy use, and maintenance requirements (Sullivan et al., 2010). Although mechanical filtration is relatively simple and immediately effective, performance can decline through screen clogging, inadequate backwashing, particle fragmentation, and poor matching between filter design and particle characteristics. Its applicability is more limited in ponds and cage systems, whereas in RAS it remains essential for maintaining downstream treatment performance and overall biosafety. Overall, conventional technologies remain essential components of water prophylaxis and biosafety. However, increasing attention is being directed toward emerging approaches that complement treatment through enhanced monitoring, surveillance, diagnostics, and preventive interventions.

## 4 Emerging approaches for sustainability

### 4.1 Water treatment

While conventional treatment methods remain essential, increasing environmental pressures and tightening regulatory frameworks have accelerated the development of more advanced and sustainable technologies (Tolkou and Kyzas, 2024). These sustainability-oriented approaches aim to enhance treatment efficiency, reduce chemical dependency, improve animal health management, and promote resource recovery within aquaculture systems (Figure 4). Their technological readiness varies considerably, ranging from established solutions already implemented in commercial aquaculture to emerging and experimental technologies that remain under active development and validation. Figure 4 summarizes the relative readiness of the principal approaches discussed in this section, including water treatment technologies, environmental nucleic acid monitoring, probiotics, phage therapy, smart sensors, and droplet microfluidic platforms. Among water treatment technologies, advanced oxidation processes (AOPs) (Ni et al., 2024), membrane bioreactors (MBRs) (Pervez et al., 2020), electrocoagulation (Boinpally et al., 2023), nanomaterial-based filtration (Khdair et al., 2025; Singh et al., 2022), and microbial fuel cells (MFCs) (Fan et al., 2023) have garnered growing interest due to their high removal efficiencies and compact system designs. However, these technologies differ in readiness for routine aquaculture use. Biofloc and IMTA-related nutrient recovery are already established in some systems (TRL 9-8; Figure 4), whereas MBRs and AOPs are mainly pilot-scale or early commercial options for intensive land-based systems and RAS (TRL 7-6; Figure 4). Electrocoagulation, nanomaterial-based filtration, and microbial fuel cells remain largely emerging (and in some cases experimental) for routine finfish aquaculture (largely TRL 5-3; Figure 4).

While these emerging technologies are often discussed across different aquaculture sectors, their relevance and performance must be interpreted within the context of finfish production systems. Intensive finfish aquaculture is characterized by high organic and nitrogenous loading, creating a need for efficient solids removal, microbial control, and stable nitrogen transformation processes (Badiola et al., 2012; Hamdhani et al., 2020). In high-intensity systems such as RAS, where water reuse and biosecurity are critical, membrane bioreactors (MBRs), advanced oxidation processes (AOPs), and biofloc systems play a central role (Davidson et al., 2011). By comparison, other aquaculture sectors, including shellfish and macroalgae production, operate under fundamentally different ecological conditions, relying on filter feeding or direct nutrient uptake. These systems depend more on natural processes and generally require less intensive water treatment (Buck et al., 2018). While such systems can offer useful functional insights, their underlying dynamics differ substantially from those of finfish production. The evaluation and application of emerging water treatment technologies here are therefore framed within the specific constraints, system designs, and biosecurity requirements of finfish aquaculture. Constructed wetlands, for example, have demonstrated significant success in removing nitrogen compounds, with studies reporting NH_4_^+^-N removal rates ranging between 86%–98% and NO_2_^–^-N removal efficiencies exceeding 99% (Crab et al., 2007). RAS is another widely adopted approach, characterized by its minimal water replacement rates (typically less than 10% daily), which contribute to reduced water consumption and controlled effluent discharge. AOPs, such as ozonation and UV/H_2_O_2_ treatment, are increasingly being explored for their efficacy in degrading persistent organic pollutants and inactivating pathogenic microorganisms (Lin et al., 2002). Membrane-based technologies, including MBRs, ultrafiltration, and reverse osmosis, offer high-efficiency separation of suspended solids, bacteria, and nutrients, although they are often associated with higher energy and maintenance costs. Media-filled bed systems and floating raft systems, especially when integrated into coupled aquaponic systems, have shown potential in reducing energy demands and nutrient loads, making them suitable for sustainable aquaculture operations (Chiam and Sarbatly, 2011).

These novel technologies offer promising alternatives to traditional systems, and especially in intensive aquaculture operations where conventional methods may fall short in managing complex waste streams (Badiola et al., 2012). For instance, MBRs integrate biological treatment with membrane filtration, producing high-quality effluent with significantly reduced pathogen loads. AOPs and electrocoagulation have demonstrated effective removal of persistent organic pollutants, including residual antibiotics and pharmaceuticals, which are often inadequately addressed by standard filtration or sedimentation (Wang et al., 2024). Additionally, microbial fuel cells offer the dual benefit of wastewater treatment and renewable energy generation by converting organic matter into electricity, pointing toward energy-neutral aquaculture solutions. Recent research has also highlighted the potential of strategies to exploit biological elements, such as biofloc technology and IMTA, which can improve both ecological sustainability and economic output (Yu et al., 2023; Chen et al., 2023; Jamal et al., 2020; Khanjani et al., 2022). Biofloc systems rely on complex microbial consortia dominated by heterotrophic bacteria (often including *Bacillus* spp. and other Proteobacteria members) that assimilate nitrogenous waste into microbial biomass, which can subsequently be used as feed, thereby reducing nutrient discharge and feed costs (Padeniya et al., 2022; Chen et al., 2023). IMTA, on the other hand, combines species from different trophic levels (typically finfish, shellfish, and macroalgae) to optimize nutrient utilization and minimize environmental impact by mimicking natural ecosystem functions (Rusco et al., 2024; Khanjani et al., 2022). These approaches not only reduce waste but also enhance overall productivity through system integration and nutrient cycling.

Despite their advantages, the adoption and scalability of emerging technologies remain constrained by high capital investment, operational complexity, and the need for technical expertise. Moreover, their applicability varies depending on aquaculture type and system scale (Li C. et al., 2025). High-tech solutions like MBRs are better suited for land-based, closed-loop systems such as RAS, whereas low-intensity operations such as seaweed or shellfish farms may not require such advanced interventions (Raza et al., 2025). Therefore, integrating these technologies into aquaculture operations must be guided by a holistic, site-specific evaluation balancing ecological benefits with economic feasibility and operational practicality (Yusoff et al., 2024; Food and Agriculture Organization of the United Nations [FAO], 2024). Thus, selection should depend on system type, treatment target, cost, operational capacity, and biosafety risk. While these approaches focus primarily on treatment and resource recovery, effective water prophylaxis also depends on technologies capable of detecting emerging risks and supporting early intervention.

### 4.2 Environmental DNA/RNA (eDNA/eRNA)

Environmental DNA (eDNA) and environmental RNA (eRNA), collectively referred to as environmental nucleic acids (eNA), offer transformative potential for aquaculture by enabling non-invasive, rapid, and scalable monitoring of aquatic organisms and their associated ecological processes. In practice, eNA can be used to detect early signals of pathogen outbreaks, monitor stock health using host- and pathogen-derived nucleic acids, and assess biodiversity and community structure within culture systems (Mérou et al., 2020; Marana et al., 2024; Kang et al., 2025; Miyata et al., 2025a). These approaches support risk assessment by tracing the presence and relative abundance of target species, invasive organisms, and commensal microbiomes, informing management decisions such as biosafety interventions, stocking strategies, and feed optimization. The growing number of field validation studies and the successful application of environmental nucleic acid monitoring across multiple pathogen groups indicate that eNA technologies have progressed beyond proof-of-concept and laboratory validation, reaching a pilot stage of development in aquaculture (TRL 7-6; Figure 4).

Moreover, eRNA, by targeting actively expressed genes, can provide functional insights into stress responses, metabolic status, immune activation, and pathogen activity, potentially enabling real-time or near-real-time management adjustments and early warning systems for sublethal perturbations that precede overt disease (Stevens and Parsley, 2023; Miyata et al., 2025b). The integration of eNA data with conventional aquaculture metrics, such as water quality parameters, growth performance, feed conversion ratios, and clinical health assessments, facilitates a holistic, systems-level understanding of culture health and resilience. These methods also enable longitudinal monitoring across production cycles, supporting trend analysis, provenance verification of stock, and environmental impact assessment. From a methodological perspective, challenges remain, including contamination control, standardization of sampling protocols (e.g., filtration volumes, preservation methods, and extraction techniques), normalization of quantitative signals, and robust interpretation of signal-to-noise ratios in complex, biodiverse confined systems (Benedicenti et al., 2024). Ongoing advances in assay design (e.g., multiplex qPCR/dPCR, targeted sequencing, and metagenomic/eRNA approaches), reference database curation, and integrative data platforms (combining omics with environmental metadata and geospatial context) are progressively enhancing the reliability, sensitivity, and cost-effectiveness of eNA-based monitoring (Dahlquist et al., 2025). Complementing these developments, CRISPR-based assays combined with isothermal amplification (e.g., RPA-Cas12a) are further expanding detection capabilities (D’Agnese et al., 2025), enabling rapid, sensitive, and potentially field-deployable detection of pathogens such as *Renibacterium salmoninarum* directly from environmental samples and supporting early-warning surveillance. These capabilities make eNA approaches highly suitable for early-warning surveillance and risk assessment within integrated biosafety decision-support frameworks (Figure 3). In parallel, applications including early detection of viral, bacterial, and parasitic threats; evaluation of feed additives and probiotic efficacy via microbial and host transcript signatures; and traceability of provenance and lineage through environmental nucleic acid profiling hold promise for more proactive, precise, and sustainable aquaculture management.

In the context of disease risk monitoring in finfish aquaculture, eNA approaches enable efficient detection and quantification of diverse pathogens, including bacteria, viruses, and parasites, without reliance on resource-intensive traditional methods such as culturing or host necropsy (Gomes et al., 2017; Bohara et al., 2022; Benedicenti et al., 2024; Marana et al., 2024; Zarantonello et al., 2026). Benedicenti et al. (2024) developed a faster, non-invasive method for pathogen surveillance in aquaculture by detecting eDNA/RNA in water rather than sampling fish, aligning with animal welfare (3Rs) principles. They showed that seawater sampling can enable earlier detection of viruses like SAV compared to traditional fish-based surveillance, but existing methods are logistically challenging due to slow filtration and cold-storage requirements. The researchers optimized a more practical workflow by demonstrating that higher filtration flow rates do not reduce detection accuracy, enabling quicker and more cost-effective sampling, and by identifying DNA/RNA Shield™ as a robust, temperature-stable buffer suitable for preserving a wide range of pathogens. They also propose a “sandwich” filtration method combining glass fiber and membrane filters to handle large water volumes and improve detection of low-abundance pathogens. Their study presents a scalable, field-friendly approach for early pathogen surveillance that could improve fish health management and reduce reliance on invasive sampling (Benedicenti et al., 2024). Together, these advances in sampling, preservation, and detection workflows demonstrate increasing operational readiness and support the classification of eNA monitoring as a pilot-stage technology for aquaculture applications (TRL 7-6; Figure 4).

In a recent study, Li (2025) provided ample evidence for the growing role of environmental nucleic acid (eNA) tools in aquaculture disease monitoring and ecosystem health assessment. The author reported that a high-throughput qPCR chip capable of simultaneously detecting 48 infectious agents, including *Aeromonas salmonicida*, *A. hydrophila*, *Vibrio anguillarum*, VHSV, ISAV, *Ichthyophthirius multifiliis*, and *Saprolegnia* sp., as well as off-flavor organisms, demonstrated high sensitivity and scalability across diverse environments. In addition, the author demonstrated that eRNA is a reliable, non-invasive indicator of fish health, reflecting host gene expression linked to stress, immunity, and infection, and that specific biomarkers have been identified for disease detection. Field validation confirmed its practical use, though results varied by location and pathogen, indicating the need for tailored biomarker panels. This work also demonstrated that eNA-based approaches can transform aquaculture monitoring by enabling rapid pathogen detection, health assessment, and antimicrobial resistance surveillance within a One Health framework, supporting more sustainable and integrated management practices. Nevertheless, beyond monitoring and surveillance, improving sustainability also requires proactive strategies for disease prevention and microbiome management.

### 4.3 Probiotics

Within the framework of non-antibiotic strategies for disease control, probiotics have received considerable attention as functional feed or additives. Probiotics, together with prebiotics and synbiotics, are widely explored due to their capacity to enhance the immune responses and growth performance (Sayes et al., 2018). They are generally considered microbial feed additives that confer benefits to the host and modulate intestinal microbiota; however, broader definitions that include live and dead organisms, inactivated cells, and microbial components have also been proposed (Hoseinifar et al., 2018).

A wide range of microorganisms, including bacteria, yeasts and microalgae, has been investigated. They were typically delivered via feed or directly in water as single strains or multi-species formulations (Newaj-Fyzul et al., 2014; Van Doan et al., 2017). Among these, lactic acid bacteria (*Lactobacillus* and *Carnobacterium*) and *Bacillus* spp. are the most extensively studied groups, with multiple reports of improved disease resistance in finfish such as Nile tilapia, rainbow trout and Atlantic salmon (Merrifield and Carnevali, 2014; Hoseinifar et al., 2018). However, probiotic effects are highly strain-specific since the same strain may produce inconsistent or even contrasting outcomes depending on three factors: (i) host species; (ii) life stage (e.g., larval, juvenile or adult phases); (iii) environmental conditions (Ringø et al., 2020). This variability highlights persistent issues with reproducibility and limits the generalizability of results across studies.

The most common modes of action include pathogens’ competitive exclusion, antimicrobial compounds production and modulation of host immune responses. In finfish, these effects are most often discussed in relation to opportunistic and endemic pathogens such as *Aeromonas* spp., *Vibrio* spp., and *Flavobacterium* spp., where probiotic-mediated competitive exclusion and antimicrobial metabolite production may reduce colonization pressure at mucosal surfaces. However, these mechanisms are not universally beneficial, as introduced microorganisms may interfere with established microbial communities or alter host–microbiota interactions in unpredictable ways; up to now, little attention has been given to how probiotics affect the microbial communities of aquaculture systems (Anokyewaa et al., 2026). The stimulation of beneficial microbial populations while suppressing pathogenic ones appears as the most reported route how probiotics can reshape the microbial communities in aquaculture, but still the responses of environmental microbial communities have not been fully investigated.

From a practical perspective, delivery strategy also plays a critical role in determining efficacy. Administration via feed allows for controlled dosing and improved stability of probiotic strains, whereas water-based delivery (particularly relevant for prophylactic treatments) is subject to rapid dilution and reduced microbial persistence (Greeshma et al., 2021). These factors can significantly affect treatment efficacy, being a major constraint for probiotics application in water-mediated disease prevention.

Moreover, regulatory constraints represent a crucial bottleneck. Although different types of probiotics are described in the literature, their approval status depends on the jurisdiction, and within European regulatory frameworks only a limited number of strains are currently authorized for use in the aquaculture sector (European Commission, 2003). The readiness of probiotic technologies also varies among applications. Conventional probiotics based on well-characterized bacterial genera such as *Bacillus*, *Lactobacillus*, and *Carnobacterium* have already reached commercial implementation in several aquaculture systems and can be considered established technologies (TRL 9-8; Figure 4). In contrast, more advanced strategies such as strain-specific formulations, microbiome-targeted interventions, multi-species consortia, and environmentally mediated probiotic applications remain under active development and are better classified as emerging technologies (TRL 5-3; Figure 4). Thus, despite the availability of established commercial products, the reliable and system-specific implementation of probiotics as a water-prophylaxis and disease-management strategy in finfish aquaculture remains context-dependent and is best classified overall as pilot-stage technology (TRL 7-6; Figure 4). Nevertheless, the available studies collectively suggest that probiotics can shift microbial community composition toward increased dominance of beneficial taxa, reduced abundance of opportunistic pathogens, and greater overall community stability, particularly under stress conditions.

### 4.4 Phage therapy

Phage therapy represents a promising “green” alternative to antibiotics, driven by rising antimicrobial resistance and the need for more targeted approaches (Żaczek et al., 2020; Li, 2025). Although phage applications have already been explored in humans and terrestrial livestock (Żaczek et al., 2020; Pereira et al., 2022), transferring these strategies to aquatic systems remains challenging due to environmental sensitivity. For instance, bacteriophages have shown effectiveness against important fish pathogens such as *Aeromonas hydrophila*, *Vibrio harveyi*, *Vibrio anguillarum*, *Edwardsiella tarda*, and *Flavobacterium columnare*, which are responsible for significant economic losses in aquaculture systems (Kalatzis et al., 2016; Silva et al., 2014). Osmotic shock can inactivate up to 99% of phage particles (Jończyk et al., 2011; Pourtois et al., 2020), and fluctuations in salinity can severely impair phage infectivity. For example, *Vibrio cholerae* phages that are highly lytic in clinical settings were unable to infect their hosts in low-salinity freshwater, highlighting how reduced osmolarity and nutrient availability restrict phage activity (Silva and Camilli, 2019). Such environmental variability is likely to be even more pronounced in fish tanks with limited water capacity, where evaporation can rapidly alter salinity.

The multiplicity of infection (MOI) is another frequently misunderstood parameter. Reported effective MOIs vary widely, from very low values (e.g., 0.01) to extremely high inocula (>10^3^–10^4^), not because of contradiction, but because MOI reflects the ratio of phage particles to bacterial cells at the time of application and depends on treatment goals, bacterial density, adsorption efficiency, latency period, and whether active phage replication is expected after dosing (Abedon, 2016; Chen et al., 2018). In practice, low MOIs may be sufficient when phages can amplify rapidly within dense bacterial populations, whereas higher MOIs may be required for rapid suppression of acute outbreaks, low host densities, poor mixing conditions, or biofilm-associated infections. For aquaculture operators, this means that fixed universal doses are unlikely to be optimal; instead, dosing strategies should be tailored to biomass, water volume, pathogen load, and system hydrodynamics.

Therapeutic success further depends on the ratio of infecting phage particles to bacterial cells, as this is crucial for effective pathogen elimination. However, studies report highly variable effective MOIs across systems, ranging from >10 for *Vibrio alginolyticus* (Kalatzis et al., 2016) to 10,000 for *Aeromonas salmonicida* in rainbow trout (Kim et al., 2013), while others observe strong lytic activity at much lower MOIs (0.01) (Chen et al., 2018). These discrepancies reflect the need to tailor phage treatments according to bacterial densities to obtain successful phage replication. In fish and seafood hatcheries, such conditions may differ significantly from those observed in wild waters (Muniesa and Jofre, 2004; Alonso-Sáez et al., 2018; Nilsson et al., 2019).

A further practical limitation is the emergence of phage-resistant bacterial mutants. As with antibiotic resistance, bacteria may evade phage infection through receptor modification, extracellular matrix production, CRISPR-Cas systems, or abortive infection mechanisms (Labrie et al., 2010; Kortright et al., 2019). Resistance can arise rapidly during monophage treatment, especially under high bacterial densities typical of intensive aquaculture. Mitigation strategies include the use of phage cocktails targeting multiple receptors, rotation of phage preparations, and combination therapy with antibiotics, probiotics, or immune stimulants (Chan et al., 2016; Kortright et al., 2019). Importantly, resistance may sometimes impose fitness costs that reduce bacterial virulence, but this outcome is not guaranteed and should be verified experimentally.

The environmental fate of therapeutic phages must also be considered in relation to standard biosecurity measures. UV irradiation and ozonation, commonly used for water disinfection in hatcheries and RAS, are highly effective against viruses and would also reduce concentrations of intentionally applied phages (Summerfelt, 2003; Muniesa et al., 2013). This creates a direct operational conflict: water treatment designed to suppress pathogens may simultaneously inactivate therapeutic phages. Consequently, successful implementation may require modified treatment schedules, localized delivery (e.g., feed coating or bath treatment), temporary bypass of UV loops, or repeated dosing after disinfection steps.

Host factors additionally influence therapeutic outcomes. Immunocompromised animals may clear infections less efficiently even when bacterial loads are reduced, whereas healthy mucosal barriers can synergize with phage activity (Łusiak-Szelachowska et al., 2014; Żaczek et al., 2020). Naturally occurring phages are also components of the fish gut and skin microbiome, where they may contribute to microbial homeostasis and pathogen exclusion (Prabhakar et al., 2025). Thus, prophylactic phage use may operate through several mechanisms: (i) direct lysis of pathogens, (ii) modulation of microbial communities at mucosal surfaces, and (iii) selection for less virulent bacterial phenotypes. Although evidence in aquaculture species remains limited, emerging studies of fish gut and skin viromes suggest that naturally occurring phages contribute to microbiome homeostasis by regulating bacterial population dynamics, constraining opportunistic taxa, and supporting microbial diversity under fluctuating environmental conditions. This indicates that phage therapy may exert broader ecological effects beyond pathogen removal, potentially influencing the overall stability and resilience of host-associated microbial communities.

Finally, regulatory uncertainty remains a major barrier to commercialization. In the European Union and many international markets, no harmonized veterinary framework specifically addresses phage therapy for aquaculture species, and products may be regulated variably as veterinary medicinal products, biological agents, or feed/water additives depending on formulation and claims (Pirnay et al., 2015; Verbeken et al., 2012). Requirements for genomic characterization, absence of lysogenic or toxin genes, manufacturing consistency, environmental risk assessment, and residue safety may substantially delay approval. Therefore, despite strong scientific interest, phage therapy remains an emerging technology (TRL 5-3; Figure 4) and routine deployment in aquaculture will depend not only on efficacy but also on clear regulatory pathways and standardized quality-control criteria. Within integrated biosafety programs, phage therapy is most likely to be implemented as a targeted intervention following pathogen detection, diagnosis, and risk assessment (Figure 3).

### 4.5 Smart sensors

Any strategy for water quality management relies on reliable monitoring and diagnostic capabilities to be effective. Ideally, diagnostic tools should be simple and inexpensive enough to enable repeated measurements, with the ultimate goal of automated and continuous operation. Point-of-care sensors have the potential to enable systemic monitoring of water quality because they can detect, and ideally quantify, key indicators that reflect the condition of a specific water body directly *in situ*. However, water quality assessment is complex, as it includes a wide range of target analytes such as whole cells (e.g., bacteria or algae), proteins (e.g., enzymes or toxins), nucleic acids (DNA/RNA from pathogens), and small molecules (e.g., nutrients, antibiotics, or heavy metals) (Fdez-Sanromán et al., 2025). While laboratory-based methods such as PCR, immunoassays, and mass spectrometry are well-established for detecting these targets, they are often impractical for continuous, on-site, or real-time monitoring in aquaculture systems. To address this gap, biosensors and microfluidic technologies (often integrated) have emerged as compact, sensitive, and cost-effective alternatives suited for both laboratory usage and field deployment (Aliazizi et al., 2024; Zhang et al., 2024; Fdez-Sanromán et al., 2025). Their performance depends on key factors such as selectivity, sensitivity, and the ability to generate interpretable outputs that can be directly used for decision-making, even by operators with limited technical expertise (Crivillé-Tena et al., 2023; Fdez-Sanromán et al., 2025; Shi et al., 2025). Although commercially available solutions remain limited, understanding the current state of the art is important for anticipating the integration of these technologies into future aquaculture practices. Smart sensors are expected to serve as frontline monitoring tools that trigger subsequent diagnostic, risk assessment, and intervention measures within integrated biosafety decision-support pathways (Figure 3).

Biosensors are typically classified according to the type of biorecognition element and the transduction method used. Biorecognition elements include (i) enzymes (e.g., urease, nitrate reductase) for detecting small molecules, (ii) antibodies for proteins and toxins, (iii) nucleic acid probes (genosensors) for microbial DNA/RNA, and (iv) whole-cell systems, including genetically engineered microorganisms that respond to bioavailable contaminants like heavy metals (Fdez-Sanromán et al., 2025). The transduction mechanism refers to how the biological event is translated into a signal. The most widely used in aquaculture are electrochemical biosensors, which detect changes in current or potential caused by biochemical reactions on an electrode surface. These sensors are ideal for *in-situ* monitoring of ions, antibiotics, and heavy metals due to their compact design and low power requirements (Shi et al., 2025). Optical biosensors, which use fluorescence, absorbance, or surface-enhanced Raman scattering (SERS), are valued for their high sensitivity and visual readout, especially in colorimetric assays or photonic platforms (Fdez-Sanromán et al., 2025; Shi et al., 2025). Other approaches, such as piezoelectric and calorimetric sensors, are less common but remain relevant in specific niche applications.

In aquaculture, biosensors could be especially useful for pathogen detection and contaminant monitoring. Genosensors have especially been used to detect pathogens. For example, Knoben et al. (2024) developed a photonic genosensor for pathogen-specific DNA detection of *Aeromonas salmonicida*, *Vagococcus salmoninarum*, and *Yersinia ruckeri* with qPCR confirmation. The LD50 for *Aeromonas salmonicida* is 3 CFU per 30–40 g fish and the lethal doses of *Vagococcus salmoninarum* are considered around 10^9^ CFU/fish. The platform successfully detected the *vapA* biomarker of *A. salmonicida*, but no numerical values like copies/μL or CFU equivalents were provided and no exact limits of detection for each pathogen were given. However, because quantitative detection limits and pathogen-specific sensitivity thresholds were not fully reported, the practical performance of the platform under aquaculture conditions remains to be further validated. Emerging approaches are increasingly moving from laboratory development toward practical aquaculture applications. For instance, a portable electrochemical immunosensor suitable for the detection of *Aeromonas salmonicida* detected the bacterium in seawater with a limit of detection (LOD) of 1 CFU/mL and a working range up to 107 CFU/mL (Messaoud et al., 2022). Similarly, a paper-based nanoparticle immunoassay has been developed for *Vibrio parahaemolyticus* in oyster hemolymph, demonstrating the growing feasibility of rapid and field-deployable pathogen diagnostics in aquaculture systems (Rodriguez-Quijada et al., 2020). Recent advances have also introduced CRISPR-based genosensors, which couple programmable nucleic acid recognition with enzymatic signal amplification. For example, Huang et al. (2023) developed a portable RPA-CRISPR/Cas12a platform in which isothermal recombinase polymerase amplification is integrated with Cas12a trans-cleavage activity, enabling highly sensitive detection down to approximately 1 copy/μL with dual visual readouts (fluorescence and lateral flow) in a closed one-pot assay within 1 h. However, despite their high analytical performance, such systems remain largely validated under controlled conditions and their robustness in complex aquaculture matrices is still unclear (TRL 5-3; Figure 4). Immunosensors can detect key metabolites, useful for contamination detection, such as the recently developed sensor that uses carbon dots as fluorescent labels for rapid, accurate detection of leucomalachite green (a derivative of the frequently used chemical malachite green) (Yoohanngoh et al., 2025). Whole-cell biosensors have also been used to detect quorum-sensing molecules such as acyl-homoserine lactones from pathogens like *Pseudomonas aeruginosa* and *Burkholderia pseudomallei* with LOD of 1 nM, that is at least 100 times lower than concentrations corresponding to virulent populations (Wu et al., 2021). Enzyme-based electrochemical sensors and aptamer-based biosensors have been developed for detecting antibiotics (e.g., oxytetracycline, sulfamethoxazole) and endocrine-disrupting chemicals with high specificity and a linear dynamic range between 0.1 and 100 μM (Zhang et al., 2024). These prototypes showed elevated performance in lab-controlled conditions, but their implementation in aquaculture requires solving several issues. Despite their potential, biosensors often face practical challenges such as sensor fouling, calibration drift, and performance degradation in complex sample matrices with organic debris, plankton, or suspended solids (Fdez-Sanromán et al., 2025). Microfluidic technologies have emerged as a powerful complementary tool, addressing many of these limitations by offering enhanced fluid control, miniaturization, and integration capabilities.

Microfluidic technology, especially when applied to lab-on-a-chip (LOC) devices, enables precise manipulation of small fluid volumes within compact, often disposable platforms. Commonly fabricated from PDMS, glass, or thermoplastics, these systems integrate sample handling, analysis, and signal output on a single chip (Pattanayak et al., 2021). Their advantages include reduced reagent consumption, high analytical sensitivity, and suitability for real-time, on-site use in many diverse environments, including aquaculture. Fabrication advances such as soft lithography, 3D printing, and paper-based microfluidics (μPADs) have lowered production costs and improved field readiness (Berlanda et al., 2020; Alexandre-Franco et al., 2024; Xu et al., 2025). μPADs are mainly attractive due to their capillary-driven flow, which eliminates the need for external pumps and supports simple visual or electrochemical readouts (Fdez-Sanromán et al., 2025).

Point-of-care (POC) biosensors frequently integrate microfluidics with binding elements (e.g., antibodies, aptamers, or protein scaffolds) and transduction techniques such as lateral flow, colorimetry, or electrochemistry. These platforms can produce results directly interpretable via smartphones (Boonkaew et al., 2024; Udhani et al., 2025), supporting remote diagnostics and decentralized monitoring. Microfluidic and electrochemical sensing platforms have been widely used to monitor ecological indicators such as toxic algae, pharmaceuticals, microplastics, and perfluorinated compounds (Oloketuyi et al., 2020; Pardeshi and Dhodapkar, 2022; Zhang et al., 2024). However, their adaptation and validation for routine aquaculture monitoring remain largely confined to laboratory-scale studies, corresponding to an emerging level of readiness (TRL 5-3; Figure 4).

Recent innovations have further expanded the capabilities of microfluidic-biosensor platforms, although these technologies remain at TRL 5-3 (Figure 4). For example, semi-circular magnetophoretic separation and nanozyme-mediated catalytic amplification have enabled *Salmonella* detection down to 41 CFU/mL within 1 h (Ding et al., 2023; Xing et al., 2023). AI integration now supports automated classification, real-time data analysis, and even microfluidic chip design optimization, improving usability and diagnostics in aquaculture environments (Fdez-Sanromán et al., 2025). In chemical monitoring, microfluidic-SERS platforms have achieved ultra-sensitive detection of aquaculture-relevant contaminants. Geosmin, which affects fish flavor and health in recirculating systems, has been detected at 0.16 ng/L within 4 min (Gong et al., 2025). Uranium ions (UO_2_^2 +^), representing heavy metal contamination, have been measured down to 7.2 × 10^–13^ M using microfluidic-SERS (He et al., 2020; Aliazizi et al., 2024). While these advances demonstrate the growing capabilities of integrated biosensor and microfluidic platforms, a distinct branch of microfluidics called droplet microfluidics has emerged as an exceptionally powerful approach for molecular diagnostics and controlled biological applications.

### 4.6 Droplet microfluidics

A unique branch of microfluidics, droplet-based microfluidics, has also shown great potential. Droplet microfluidics enables the generation and manipulation of discrete droplets, such as water-in-oil droplets, which function as isolated microreactors (Fergola et al., 2025). Compared to traditional channel-based systems, droplet microfluidics allows for ultra-high-throughput screening and compartmentalized reactions, making it highly appropriate for molecular diagnostics (Bartkova et al., 2024). One of its most impactful applications in aquaculture is digital droplet PCR (ddPCR), which offers highly sensitive detection of pathogens with LODs ranging from 0.07 to 3 copies per reaction (Sumon et al., 2024). Such sensitivity is critical for detecting low-level or latent infections, monitoring transmission routes, and identifying early-stage outbreaks that may be missed by conventional diagnostic methods. ddPCR also supports robust immune response monitoring and the evaluation of vaccine or probiotic efficacy. Consequently, droplet-based molecular diagnostics are well suited to confirmatory testing and pathogen quantification, supporting risk assessment and targeted intervention within integrated biosafety decision-support frameworks (Figure 3).

Recent studies highlight ddPCR applicability to viral pathogen detection in finfish aquaculture. Zhao et al. (2023) characterized a novel virus related to Tilapia lake virus (TiLV) from real outbreak cases, demonstrating its association with disease, although pathogenicity and quantitative infection thresholds remain uncertain. In contrast, Yin et al. (2024) developed a highly sensitive ddPCR assay for scale drop disease virus (SDDV), capable of detecting extremely low viral loads (1–2 copies/μL) with high specificity and reproducibility. While the former relied on field-collected infected fish combined with laboratory analyses, the latter demonstrated strong analytical performance and practical diagnostic applicability through validation on real fish tissue samples. Together, these studies illustrate both the diagnostic potential and current limitations of ddPCR in linking pathogen detection with disease severity in aquaculture systems. Consequently, its technology readiness currently lies between the emerging and experimental categories (TRL 5-1; Figure 4).

Emerging approaches such as CRISPR-integrated digital detection and multiplex ddPCR platforms have further expanded the analytical capabilities of droplet-based systems, offering the potential for highly sensitive and quantitative pathogen detection. However, CRISPR-ddPCR hybrid approaches remain largely at the experimental stage (TRL 2-1; Figure 4) and have not yet been applied in aquaculture monitoring systems (Sumon et al., 2024; Rieder et al., 2025).

Beyond diagnostics, droplet microfluidics shows immense promise in probiotic and prebiotic encapsulation (Yang et al., 2024; Wang et al., 2024), an emerging strategy in finfish aquaculture prophylaxis (Dezfooli et al., 2019). Especially the capability of generating alginate microparticles via droplet microfluidics is desirable due to the natural origin, non-toxicity, and gentle gelation, making them ideal for preserving microbial viability and enabling controlled release in aquatic environments (Zhang et al., 2021). Moreover, compatibility with droplet microfluidics allows for the precise production of uniform capsules, which is critical for consistent dosing and effective gut colonization. This combination of properties makes alginate-based systems attractive for sustainable, antibiotic-free aquaculture practices (Zhang et al., 2021). However, despite its potential, droplet microfluidics still faces the same general challenges in scaling up production and customizing release kinetics under aquatic conditions, thus remaining in the experimental stage as an application tool for probiotic and prebiotic encapsulation in aquaculture (TRL 2-1; Figure 4). Future development should focus on refining encapsulation systems for aquatic use, enabling eco-friendly and effective delivery of functional feed additives (Dezfooli et al., 2019).

**FIGURE 5:**
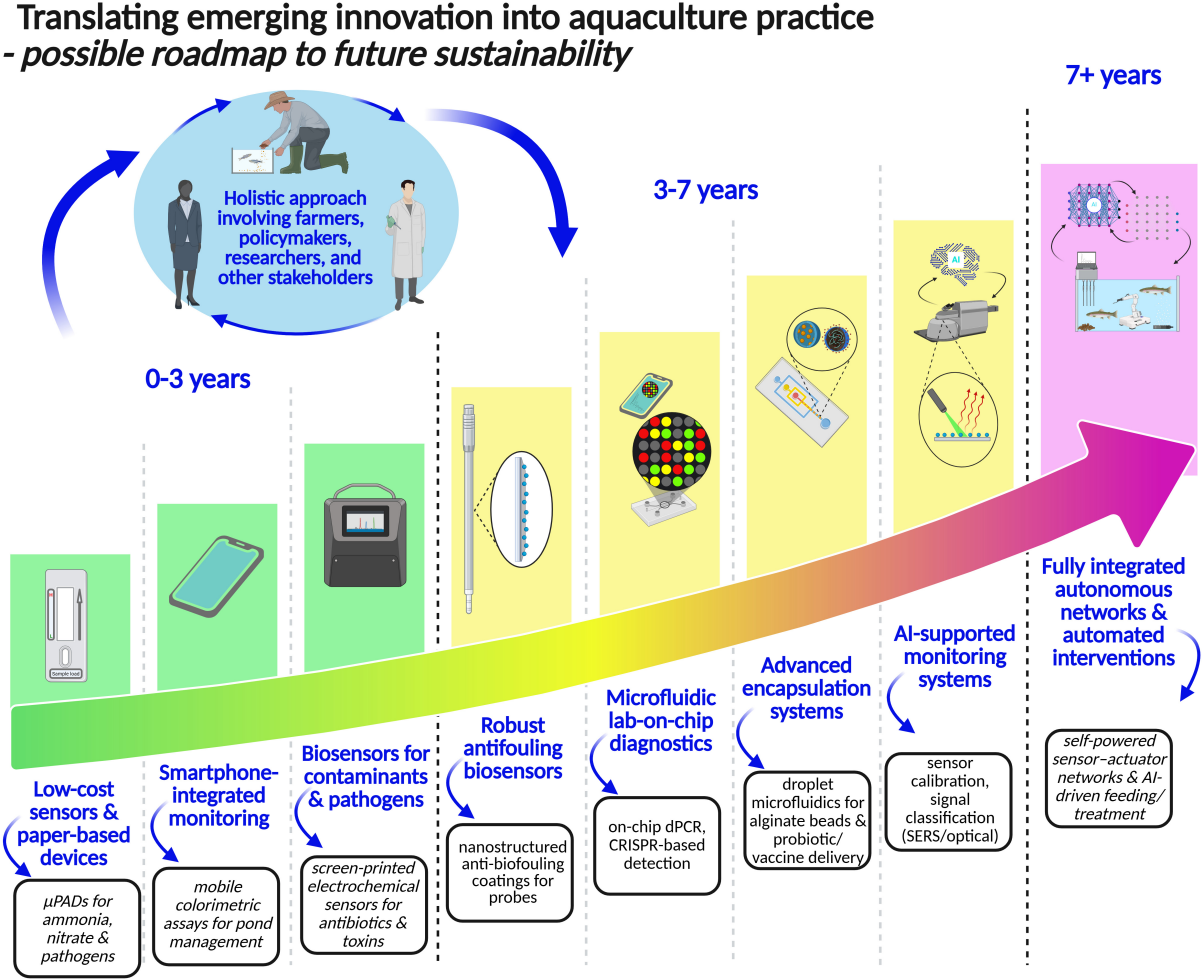
Schematic overview and examples of envisioned potential technological innovations for enabling future finfish aquaculture sustainability. 0–3 years emphasizes deployable μPADs, smartphone biosensors, and electrochemical readouts; 3–7 years target robust antifouling sensors, ddPCR/CRISPR diagnostics, and microfluidic encapsulation; 7+ year prediction envisions fully autonomous, AI-integrated sensor networks for proactive water management. All innovations and implementations need a holistic approach with constant communication among researchers, producers, policymakers, and other stakeholders. Created in BioRender. Bartkova, S. (2026) https://BioRender.com/fwtdtej.

As highlighted by Yu Q. et al. (2025), microfluidic technology is rapidly transitioning from lab-scale innovation to real-world marine applications. With continued advancements in integrated sensing, miniaturization, and automation, microfluidics could become a cornerstone of sustainable aquaculture diagnostics and prophylaxis (Yu Q. et al., 2025; Aliazizi et al., 2024; AlMashrea et al., 2024; Rieder et al., 2025). Nevertheless, realizing its full potential requires tailoring solutions to the diversity of aquaculture practices. For example, RAS demands highly sensitive, continuous monitoring of metabolites, microbial dynamics, and off-flavor compounds, whereas open-sea cages may benefit more from portable and robust biosensors capable of detecting pathogens and harmful algal blooms in real time. In pond-based systems, where resource constraints are more common, low-cost, paper-based or smartphone-integrated assays could enable individual monitoring by farmers with minimal technical training. Overall, optimizing robustness under variable water conditions (Aliazizi et al., 2024), ensuring compatibility with complex sample matrices (Abdelhamid et al., 2025), and developing user-friendly interfaces for non-specialist operators (Lai et al., 2022; Loima et al., 2025) remain central challenges that must be addressed for successful implementation. Beyond sensing, the application of microfluidics for encapsulation and controlled release of probiotics or vaccines also requires system-specific refinement. In intensive RAS, uniform and predictable release profiles may be critical to stabilize microbiota and reduce antibiotic dependency, whereas in open-water farming, scalable production and resistance to environmental fluctuations (e.g., salinity, temperature, turbulence) are essential (Tammas et al., 2024). Ultimately, future progress will rely on interdisciplinary collaboration between engineers, biologists, and industry stakeholders to co-design solutions that are not only reliable, sensitive, and portable, but also tailored to the operational realities of different aquaculture systems (Lasner and Gimpel, 2024). Integration with artificial intelligence (AI), including AI of things (AioT) and machine learning models (MLMs), will be essential to compensate for possible operator data misinterpretation and optimize large-scale operations (Bonnichsen et al., 2025; Fini et al., 2025; Huang and Khabusi, 2025; Figure 5).

## 5 Conclusion

Effective water quality management, water prophylaxis, and biosafety are fundamental to the sustainability, resilience, and long-term productivity of finfish aquaculture. As production systems become increasingly intensive and environmental pressures continue to grow, disease prevention can no longer rely solely on maintaining physicochemical water-quality parameters. Instead, it requires an integrated understanding of how water quality, microbial ecology, pathogen dynamics, antimicrobial resistance, and management practices interact to influence fish health and disease risk.

This review highlights that conventional technologies, including mechanical filtration, biofiltration, ultraviolet disinfection, and ozonation, remain the foundation of water prophylaxis in modern aquaculture systems. However, emerging approaches such as eNA monitoring, probiotics, phage therapy, biosensors, smart sensors, and microfluidic platforms are expanding opportunities for earlier pathogen detection, improved risk assessment, and more targeted interventions. As illustrated by the technology-readiness framework presented in Figure 4, these approaches differ substantially in maturity, ranging from established technologies already integrated into commercial production systems to experimental concepts that require further validation before routine implementation.

A key conclusion of this review is that effective biosafety management depends increasingly on the integration of monitoring, surveillance, diagnostics, risk assessment, and intervention measures rather than on individual technologies operating independently. The decision-support framework proposed in Figure 3 demonstrates how environmental signals, pathogen surveillance, molecular diagnostics, biological interventions, and water-treatment strategies can be combined into a coordinated, risk-based approach to disease prevention across diverse finfish production systems.

Future developments are likely to focus on increasing precision, automation, and real-time decision-making capacity through advances in biosensors, environmental nucleic acid technologies, microfluidics, artificial intelligence, and integrated data platforms. Nevertheless, successful implementation will depend on improving robustness under real-world production conditions, reducing costs, increasing scalability, and ensuring compatibility with diverse aquaculture environments. Biological prophylaxis strategies such as probiotics and phage therapy will likewise require greater standardization, ecological validation, and regulatory harmonization before their full potential can be realized. At the same time, improving our understanding of the interactions among water quality, microbiome stability, pathogen dynamics, and antimicrobial resistance will remain essential for developing resilient aquaculture systems under changing environmental conditions.

The innovation roadmap presented in Figure 5 illustrates one possible pathway through which emerging technologies may progressively transition from laboratory development to routine aquaculture practice. However, technological innovation alone will not be sufficient. Sustainable implementation will require a holistic approach involving farmers, researchers, industry stakeholders, policymakers, and regulators working together to translate scientific advances into practical and economically viable solutions.

Ultimately, the future of finfish aquaculture will depend on integrating conventional water-treatment infrastructure, advanced monitoring technologies, biological interventions, and evidence-based management within a unified framework of water prophylaxis and biosafety. Such an approach offers the greatest potential to improve fish health and welfare, reduce environmental impacts, strengthen antimicrobial stewardship, and support aquaculture systems that are productive, resilient, and aligned with One Health principles.
